# AtROS1 overexpression provides evidence for epigenetic regulation of genes encoding enzymes of flavonoid biosynthesis and antioxidant pathways during salt stress in transgenic tobacco

**DOI:** 10.1093/jxb/erv304

**Published:** 2015-06-25

**Authors:** Poonam Bharti, Monika Mahajan, Ajay K. Vishwakarma, Jyoti Bhardwaj, Sudesh Kumar Yadav

**Affiliations:** Biotechnology Division, CSIR-Institute of Himalayan Bioresource Technology, Palampur 176061, Himanchal Pradesh, India

**Keywords:** Epigenetic regulation, coding regions, methylation status, promoters, ROS1 overexpression, salt stress, tobacco.

## Abstract

In plants, epigenetic changes have been identified as regulators of developmental events during normal growth as well as environmental stress exposures. Flavonoid biosynthetic and antioxidant pathways play a significant role in plant defence during their exposure to environmental cues. The aim of this study was to unravel whether genes encoding enzymes of flavonoid biosynthetic and antioxidant pathways are under epigenetic regulation, particularly DNA methylation, during salt stress. For this, a repressor of silencing from Arabidopsis, *AtROS1*, was overexpressed in transgenic tobacco. Generated transgenics were evaluated to examine the influence of *AtROS1* on methylation status of promoters as well as on coding regions of genes encoding enzymes of flavonoids biosynthesis and antioxidant pathways. Overexpression of *AtROS1* increases the demethylation levels of both promoters as well as coding regions of genes encoding chalcone synthase, chalcone isomerase, flavanone 3-hydroxylase, flavonol synthase, dihydroflavonol 4-reductase, and anthocyanidin synthase of the flavonoid biosynthetic pathway, and glutathione S-transferase, ascorbate peroxidase, glutathione peroxidase, and glutathione reductase of the antioxidant pathway during control conditions. The level of demethylation was further increased at promoters as well as coding regions of these genes during salt-stress conditions. Transgenic tobacco overexpressing *AtROS1* showed tolerance to salt stress that could have been due to the higher expression levels of the genes encoding enzymes of the flavonoid biosynthetic and antioxidant pathways. This is the first comprehensive study documenting the epigenetic regulation of flavonoid biosynthetic and antioxidant pathways during salt-stress exposure of plants.

## Highlight

*AtROS1* overexpression increases demethylation levels of promoters and coding regions of genes encoding enzymes of the flavonoid biosynthetic and antioxidant pathways to provide salt stress tolerance in transgenic tobacco.

## Introduction

Crop yield is severely affected by the presence of high sodium ions in the soil all over the world (Greenway and Munns, 1980). Salt stress influences many physiological, biochemical, cellular, and molecular processes of a plant (Jouyban, 2012). To limit the effect of salt stress, plants have developed defence mechanisms (Xiong *et al.*, 2002; Gupta and Huang, 2014). Enzymes of flavonoid and antioxidative pathways play a key role and protect the plant cells from oxidative damage by scavenging of free radicals (Bors *et al.*, 1990; Fini *et al.*, 2011; Ghasemzadeh and Ghasemzadeh, 2011; Sharma *et al.*, 2012). Expression of various genes encoding enzymes of flavonoid and antioxidative pathways is enhanced during salt stress conditions, and transgenic plants overexpressing many of the genes of these pathways are tolerant to salt stress (Shinozaki *et al.*, 1997; Roxas *et al.*, 2000; Sairam *et al.*, 2002; Rodriguez Milla *et al.*, 2003; Walia *et al.*, 2005; Lee *et al.*, 2007; Ashraf, 2009; Yang *et al.*, 2009; Gill and Tuteja, 2010; Matus *et al.*, 2010; Le Martret *et al.*, 2011; G Li *et al.*, 2013; X Li *et al.*, 2013; Singh *et al.*, 2014).

Epigenetic regulation of various cellular and molecular processes is a major player in the control of growth and development of plants (Hauser *et al.*, 2011). The expression of genes during stress is also reported to be under the regulation of chromatin-associated modifications (Henderson and Jacobsen, 2007). This is due to DNA methylation at promoter regions or blocking of transcription activators and recruitment of transcription repressors at methylated sites (Bird, 2002). A study has also disclosed that about one-third of expressed genes are methylated at coding regions in plants (Takuno and Gaut, 2012). DNA methylation is typically a cytosine methyltransferase-catalysed methylation of cytosine residues during replication, yielding 5-methylcytosine (5-mC) (Finnegan and Kovac, 2000). In mammals, cytosine methylation occurs normally at CG sequences. In plants, it is found in symmetric CG and CHG as well as asymmetric CHH sequences (where H is A, C, or T) (Furner and Matzke, 2011). Plants have at least three classes of cytosine methyltransferases, which differ in their protein structure and function (Finnegan and Kovac, 2000; Bird, 2002). Some stress-induced cytosine methylation responses are retained in the plants as stress memory and may be inherited across generations (Chinnusamy and Zhu, 2009). However, removal of methylated cytosine occurs either passively during replication cycles and/or actively via an enzymatic mechanism. In plants, active DNA demethylation is carried out by DNA glycosylases belonging to the DEMETER (DME) family. The DME family consists of a group of proteins such as DME, DML2, DML3, and REPRESSOR OF SILENCING 1 (ROS1) DNA glycosylases. DME is required for genome imprinting whereas demethylation by DML2, DML3, and ROS1 is involved in shielding the genome from harmful methylations and in DNA repair, similar to the HhH-GDP superfamily proteins (Choi *et al.*, 2002; Gong *et al.*, 2002; Agius *et al.*, 2006; Gehring *et al.*, 2006; Morales-Ruiz *et al.*, 2006; Penterman *et al.*, 2007*a*; Penterman *et al.*, 2007*b*; Zhu *et al.*, 2007; Zhu, 2009).

ROS1 acts as a suppressor of transcriptional gene silencing by catalysing the removal of 5-mC and demethylating target DNA without the need for replication through a base excision repair pathway (Gong *et al.*, 2002; Roldán-Arjona and Ariza, 2009). It is a 1393 amino acid–long nuclear protein. It encodes the HhH motif in an endonucleases III domain with an invariant lysine residue (lys-953), and shows bifunctional DNA glycosylase/lyase activity against 5-mC (Krokan *et al.*, 1997; Gong *et al.*, 2002; Morales-Ruiz *et al.*, 2006; Ponferrada-Marín *et al.*, 2010). A short N-terminal lysine-rich domain is conserved across the ROS1/DME family that mediates strong binding of ROS1 to DNA in a methylation-independent manner for efficient activity on 5-mC-G, but not for T-G processing (Ponferrada-Marín *et al.*, 2009; Ponferrada-Marín *et al.*, 2012). However, removal of this N-terminal domain has been reported to affect their activity. ROS1 functions constitutively during developmental stages of a plant. Analysis of the ros1 mutant determined that ROS1 functions in demethylation of several transposons and other genes. Hence, active DNA demethylation is important to keep the plant epigenome plastic so that it can efficiently respond to developmental and environmental cues (Gong *et al.*, 2002; Zhu *et al.*, 2007).

Here, by raising transgenic tobacco (*Nicotiana tabacum* L.) overexpressing ROS1 from *Arabidopsis* (AtROS1), evidence is provided for the epigenetic regulation of genes encoding enzymes of the flavonoids biosynthesis and antioxidant pathways during salt-stress exposure in tobacco plant. AtROS1-mediated demethylation enhanced the expression levels of genes encoding enzymes of these pathways and hence improved the tolerance of transgenic plants to salt stress.

## Materials and methods

### Seed germination and plant transformation

Tobacco (*Nicotiana tabacum* cv. ‘Xanthi’) seeds were surface sterilized with 10% Tween-20 for 5min, 70% ethanol for 30 s, and 0.001% HgCl_2_ for 3min. Seeds were then washed three to four times in autoclaved distilled water and germinated on 0.8% agar in Murashige and Skoog (MS) medium (Duchefa Biochemie). The plants were grown in tissue culture at 25±2ºC for 7 days or until the seeds germinated. Fifteen-day-old seedlings were transferred into flasks containing fresh MS medium and allowed to grow. The pEGAD vector containing cDNA of *AtROS1* was kindly provided by Professor Zhizhong Gong, State Key Laboratory of Plant Physiology and Biochemistry, College of Biological Sciences, China Agricultural University, Beijing, China, and was used for *Agrobacterium tumefaciens* strain LBA4404-mediated transformation of tobacco. The leaves of 35-day-old tobacco plants were subsequently used as explant for *Agrobacterium*-mediated infection through the leaf-disc transformation method. After transformation, leaf discs were incubated for 48h on plates containing MS media with growth regulators. Thereafter, leaf discs were transferred to selective medium containing MS media, cefotaxime (250 µg/ml), and carbenicillin (250 µg/ml). After one month, plantlets regenerated from callus were transferred into flasks with fresh MS medium and allowed to grow. Plants were then transferred to pots containing a mixture of garden soil, clay, and sand. Plants were kept in a greenhouse for a week to acclimatize and finally transferred into pots containing garden soil and allowed to set seeds by self-pollination.

### Identification and selection of transgenic plants

Transformants were selected by the use of Basta spray on 1-month-old plants. Genomic DNA was isolated from leaves of *AtROS1* transgenic lines and wild-type tobacco plants and used to amplify *AtROS1* gene with gene-specific primers (Supplementary Table S1). Reverse transcription polymerase chain reaction (RT-PCR) was then performed to check the expression level of *AtROS1* gene in transformed plants. For this purpose, total RNA of wild-type and transgenic lines was extracted from 100mg of leaf tissue by the IRIS method (Ghawana *et al.*, 2011) and reverse transcribed into cDNA from 1 µg of total RNA using SuperScript-III Reverse Transcriptase (Invitrogen, USA) according to manufacturer’s instructions. The resulting cDNA was used as a template for amplification of the target gene with gene-specific internal primers. The amplification of 26SrRNA was used as an internal control in the expression experiment. Among the confirmed transgenic tobacco lines, two homozygous lines that overexpressed *AtROS1*, TL-1 and TL-2 from the T3 generation, were used for further analysis.

### Salt stress tolerance analysis

To determine whether constitutive expression of *AtROS1* influenced the growth of seedlings under salt treatment, both wild-type and transgenic lines were treated with different NaCl concentrations at seedling stage. Fifteen-day-old seedlings of wild-type and transgenic lines in earthen pots were supplemented with 0mM, 50mM, 100mM, and 200mM concentrations of NaCl, at 25±2ºC. After 30 days of treatment, phenotypic variations that developed in plants were photographed. Seedling fresh weight was measured and the seedlings immediately stored in liquid nitrogen for further experiments.

### Total RNA extraction and cDNA preparation

Total RNA of NaCl-treated and control seedlings of both wild-type and transgenic tobacco plants was isolated and treated with 1U/µl of DNase-I (Thermo Scientific, USA) to remove any genomic DNA contamination. First-strand cDNA synthesis was carried out using the High Capacity cDNA Reverse Transcription Kit (Thermo Scientific) with 2 µg of purified total RNA in a final reaction volume of 20 µl. The prepared cDNA was used for quantitative real-time PCR analysis.

### Quantitative real-time PCR analysis

For the expression study of flavonoid biosynthetic and antioxidative pathway genes under normal and salt stress conditions, a quantitative real-time PCR was performed using 4.5 µl of diluted cDNA with the DyNamo Flash SYBR Green qPCR Kit (Thermo Scientific) and gene-specific primers (Supplementary Table S2) designed by PrimerExpress 3.0.1 software (Applied Biosystems, USA) to a final volume of 32 µl for each reaction. The expression analysis was carried out in triplicate by SYBR Green dye chemistry detection with an ABI 7500 Step One Plus Real-Time PCR System (Applied Biosystems). The initial stage was 94°C for 5min, followed by 45 cycling stages of 94°C for 30 s, 58–59°C for 30 s, and 72°C for 30 s (data collection), with a step and hold melt curve stage. The experiments were analysed according to baseline and a manual threshold, and data was collected with ABI 7500 System Step One v2.2.2 Software. Relative quantifications were calculated using the Ct method (comparative 2^ddCT method) and normalized with the expression level of an endogenous gene 26SrRNA.

### Nuclei isolation

Wild-type tobacco and *AtROS1* transgenic tobacco lines grown for 15 days in a greenhouse were treated with different NaCl concentrations (0mM, 50mM, 100mM, and 200mM) on every alternate day for 30 days. Nuclei of NaCl-treated and untreated wild-type and transgenic tobacco plants were isolated on day 0, day 15, and day 30 using the CelLytic PN Plant Nuclei Isolation/Extraction Kit (Sigma Aldrich), and stored immediately at −80°C for the demethylase activity assay.

### Demethylase activity assay

Demethylase activity was calorimetrically quantified through an enzyme-linked immunosorbent assay-like reaction using an ab156908 EpiSeeker DNA demethylase (total) Activity Quantification Ultra Assay Kit according to manufacturer’s instructions (Abcam). Five micrograms of nuclear extract from each sample was used and the absorbance of the developed colour measured on a microplate reader (Synergy BioTek H1 Hybrid Reader) at 450nm with an optical reference wavelength of 655nm. Total demethylase activity was calculated according to the following formula:

Demethylase activity (OD/h/mg) = [OD (Control − Blank) − OD (Sample − Blank)] / [NE amount (µg)/1000] × Hour

Where ‘Control’ was the sample wells containing demethylase assay buffer and enzyme buffer but without nuclear extracts, ‘Sample’ was the sample wells containing nuclear extracts and demethylase assay buffer, ‘Blank’ was the sample wells containing only demethylase assay buffer, NE was the amount of nuclear extract added (μg), and Hour was the incubation time of the enzymatic reaction.

### Promoter isolation

Genomic DNA of control and salt stress-treated wild-type and transgenic tobacco plants was isolated using the DNeasy Plant Mini Kit (Qiagen, USA) and purity was checked on a Nanodrop spectrophotometer. Highly pure genomic DNA was subjected to promoter isolation for genes encoding enzymes of the flavonoid biosynthesis and antioxidative pathways with the help of the Genome Walker Universal Kit (Clontech) according to the instructions. The genomic DNA was differentially digested with four blunt-end restriction enzymes, DraI, EcoRV, PvuII, and StuI, for 16–18h at 37°C to obtain four genome walker libraries. Digested genomic DNA fragments were purified by phenol, chloroform, and ethanol precipitation. After purification, digested fragments were ligated with universal adaptors provided with the kit.

Primary PCR was performed using adaptor primer (AP1) and gene-specific primer-1 (Supplementary Table S3) and high specificity Amplitaq Gold PCR master mix (Applied Biosystems). Diluted product of primary PCR (1:50) was used as a template for secondary nested PCR with a second adaptor primer (AP2) and gene-specific nested primer-2 (Supplementary Table S3). Two-step thermal cycling conditions for primary PCR were as follows: seven cycles of 94°C for 25 s and 72°C for 3min, and 37 cycles of 94°C for 25s and 67°C for 3min, with an additional final cycle of 67°C for 7min. For the nested PCR, cycles were as follows: five cycles of 94°C for 25 s and 72°C for 3min, and 25 cycles of 94°C for 25 s and 67°C for 3min, with an additional final cycle of 67°C for 7min. PCR products were checked on 1.5% agarose gel stained with ethidium bromide along with a 1 kilobase DNA ladder (Thermo Scientific). The banding patterns observed on agarose gel supposed to contain putative promoters were eluted using the Gel Elute Kit (Sigma Aldrich) and cloned into the pGEM-T Easy Vector (Promega) system. Transformants were confirmed by colony PCR and plasmids of positive clones were subjected to a sequencing reaction using the Big Dye Terminator v1.1, v3.1 Sequencing Kit (Applied Biosystems) with M13 forward and reverse primers.

### Sodium bisulfite modification, amplification, cloning, and sequencing

Promoters isolated by genome walking were subjected to bisulfite treatment. For this purpose, 500ng to 1µg of eluted DNA was treated with sodium bisulfite and later purified using the Epitect Bisulfite kit (Qiagen) according to manufacturer’s instructions. Converted DNA was stored at −20°C in aliquots. The primer pairs specific for bisulfite-converted DNA were designed by MethPrimer (Supplementary Table S4), an online tool to design primers specific for methylation mapping (Li and Dahiya, 2002) and promoter sequences as reference. Two-step touchdown PCR amplification of treated DNA was performed in a reaction mix of 50 µl for the first step, containing 2 µl of converted DNA, 10mM deoxynucleotide triphosphates, 1 µM primers, and Advantage 2 Polymerase Mix (Clontech) under the following thermal cycling conditions: 94°C for 2min; followed by 21 cycles of 94°C for 30 s, annealing temp°C+5°C for 30 s with a decrease of 0.5°C per cycle, and 72°C for 1min; then 35 cycles of 94°C for 30 s, Tm°C for 30 s, and 72°C for 1min; and a final extension at 72°C for 10min. In the second step, pre-nested PCR, 4 µl of primary PCR product was used as template in a reaction mix of 50 µl (in duplicates) for the next round of amplification under the above thermal cycling conditions. PCR products were checked on 2% agarose gel stained with ethidium bromide and 100 base pair DNA ladder (Thermo Scientific). Bands were visualized under a UV transilluminator. Amplified products were purified using a PCR Purification Kit (Norgen Biotek Corp., Canada) according to the manufacturer’s instructions and cloned into a pGEM-T Easy Vector (Promega) system. Transformants were confirmed by colony PCR and plasmids of positive clones were subjected to a sequencing reaction using the Big Dye Terminator v1.1, v3.1 Sequencing Kit (Applied Biosystems) with M13 forward and reverse primers.

### Methylation-specific PCR

Methylation-specific PCR was carried out as described earlier (Herman *et al.*, 1996). Genomic DNA (1 µg) of wild-type and transgenic lines treated with 200mM NaCl for 30 days was bisulfite-converted using the Epitect Bisulfite Kit (Qiagen) according to manufacturer’s instructions. Two primer pairs specific to bisulfite-converted DNA for methylated (Supplementary Table S5) and unmethylated (Supplementary Table S6) templates were designed by MethPrimer software (Li and Dehiya., 2002). Two-step touchdown PCR amplification of treated DNA was performed for methylated and unmethylated DNA in a reaction mix of 25 µl using the Advantage 2 polymerase mix (Clontech) as above. PCR products were checked on 1.5% agarose gel stained with ethidium bromide and visualized under a UV transilluminator.

### Data analysis

Sequencing data was analysed by Sequencher version 5.2.4 and BiQAnalyzer, an online software tool specifically designed for methylation mapping of bisulfite sequencing data (Bock *et al.*, 2005).

### Statistical analysis

Each experiment was conducted in at least three biological replicates. The data were subjected to analysis of variance, and least significant difference was calculated for probability, *P* < 0.05 and *P* < 0.01.

## Results

### Generation of *AtROS1* overexpressing transgenic tobacco plants

**Fig. 1.**
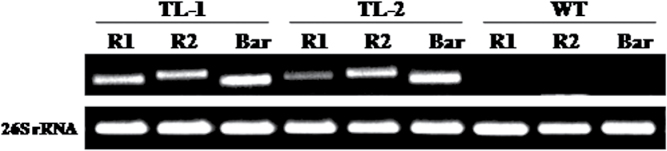
Confirmation of *AtROS1* expression in transgenic tobacco by reverse transcription PCR analysis. The expression analysis of 26SrRNA was used as internal control. R1, internal primer 1; R2, internal primer 2; Bar, Basta selection marker-specific primer.

Transgenic tobacco lines overexpressing *AtROS1* were generated (Supplementary Fig. S1A and S1B). Transgenics were selected by Basta selection and confirmed by genomic DNA PCR using transgene-specific primers (Supplementary Fig. S1C). The transcript expression of *AtROS1* in transgenic tobacco plants was conducted by semi-quantitative PCR analysis (Fig. 1). Transgenic lines were allowed to set seeds in the greenhouse. Confirmed homozygous tobacco transgenic lines TL-1 and TL-2 from the T3 generation were chosen for further analysis.

### Response of *AtROS1* transgenic tobacco during salt stress

**Fig. 2.**
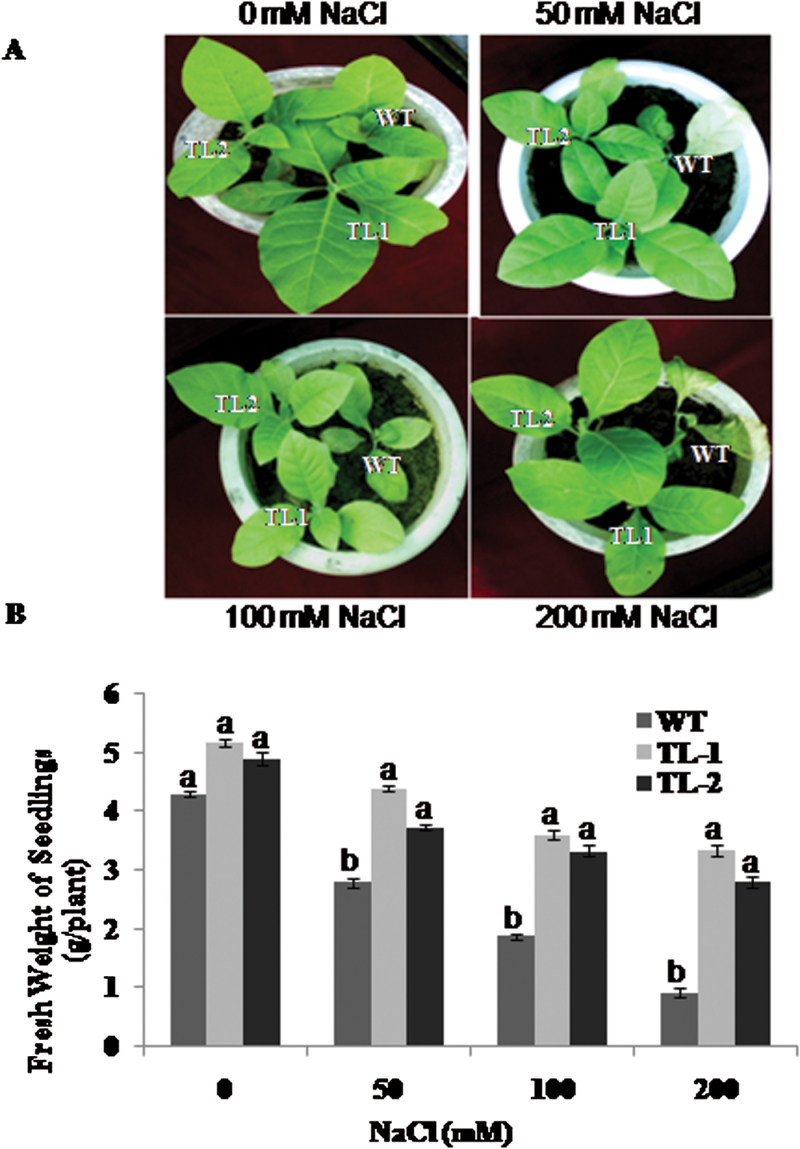
Salt-stress response of *AtROS1* transgenic tobacco plants. (A) Morphological affect of salt stress in wild-type and transgenic lines. Fifteen-day-old seedlings of wild-type and transgenic lines grown on MS medium supplemented with different NaCl concentrations for 30 days and photographed. (B) Fresh weight of wild-type and transgenic seedlings treated with different concentrations of NaCl for 30 days. Different letters on error bar represents significant differences within treatment group, i.e. data marked ‘a’ is significantly different from data marked ‘b’ with *P* < 0.05.

Fifteen-day-old seedlings of wild-type and transgenic tobacco plants were exposed to 0mM, 50mM, 100mM, and 200mM concentrations of NaCl for 30 days. Transgenic plant lines TL-1 and TL-2 was found to have better growth than wild-type plants (Fig. 2A). The control plants showed retarded growth and chlorosis with increases in NaCl exposure concentration and time. The growth of transgenic plants was not affected or less affected, with improved salt stress tolerance correlated to the overexpression of *AtROS1* in transgenic tobacco. After 30 days, plants were analysed for total fresh weight. Under control conditions, the average fresh weight of one seedling from the wild-type and the transgenic lines was not significantly different. Upon exposure of wild-type plants to 50mM, 100mM, and 200mM NaCl, total fresh weight was reduced from 4.3g to 2.8g, 1.88g, and 0.93g, respectively. The reduction in fresh weight of transgenic plants was lower, from 5.2g to 4.38g, 3.61g, and 3.35g for TL-1 and from 4.9g to 3.73g, 3.32g, and 2.79g for TL-2 during exposure to 50mM, 100mM, and 200mM NaCl, respectively (Fig. 2B). Hence, the reduction in total fresh weight was more severe in wild-type plants than in transgenic plants during salt stress.

### Influence of *AtROS1* on expression of genes encoding enzymes of flavonoid biosynthetic and antioxidant pathways in transgenic tobacco

**Fig. 3.**
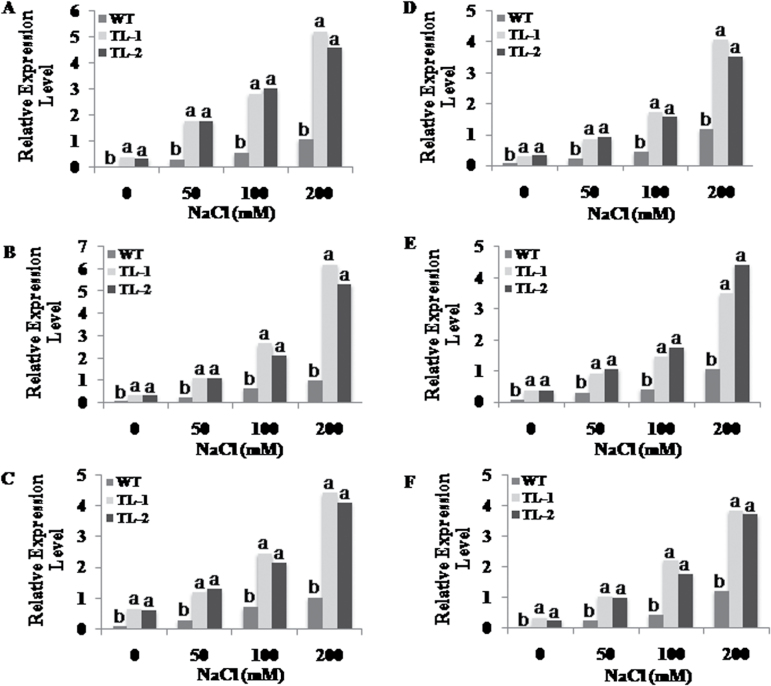
Influence of *AtROS1* overexpression on relative transcript expression of genes encoding enzymes of flavonoid biosynthetic pathway in transgenic tobacco. Relative transcript expression of genes encoding enzymes (A) CHS, (B) CHI, (C) F3H, (D) FLS, (E) DFR, and (F) ANS under normal and salt stress conditions in wild type and transgenic lines. 26SrRNA was used as internal control in the expression analyses experiment. Different letters on error bar represents significant differences within treatment group, i.e. data marked ‘a’ is significantly different from data marked ‘b’ with *P* < 0.05.

**Fig. 4.**
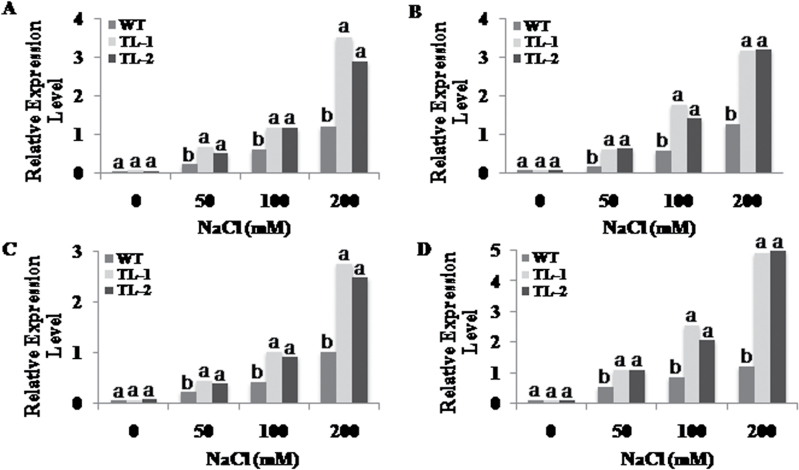
Influence of *AtROS1* overexpression on relative transcript expression of genes encoding enzymes of antioxidative system. Relative transcript expression of genes encoding enzymes (A) GST, (B) APx, (C) GPx, and (D) GR under normal and salt stress conditions in wild-type and transgenic lines. 26SrRNA was used as internal control in the expression analyses experiment. Different letters on error bar represents significant differences within treatment group, i.e. data marked ‘a’ is significantly different from data marked ‘b’ with *P* < 0.05.

To inspect the influence of demethylase activity of *AtROS1*, the expression of genes encoding chalcone synthase (CHS), chalcone isomerase (CHI), flavanone 3-hydroxylase (F3H), flavonol synthase (FLS), dihydroflavonol 4-reductase (DFR), and anthocyanidin synthase (ANS) of the flavonoid biosynthetic pathway, and glutathione S-transferase (GST), ascorbate peroxidase (APx), glutathione peroxidase (GPx), and glutathione reductase (GR) of the antioxidative pathway were analysed in 15-day-old seedlings of NaCL-treated wild-type and transgenic lines. The mRNA levels of genes encoding enzymes of the flavonoid biosynthetic pathway were higher in transgenic lines compared to wild type under control conditions. During salt stress, the expression of flavonoid biosynthetic pathway genes was upregulated in both wild-type and transgenic lines but to a higher extent in transgenic lines compared to wild-type plants. In comparison to the wild-type lines, the expression levels in transgenic plants under salt stress were up to six times higher for CHI; five times higher for CHS; and three to four times higher for F3H, FLS, DFR, and ANS (Fig. 3). Similarly, the expression levels of antioxidative pathway genes were also increased to a greater extent during salt stress (200mM NaCl) in transgenic plants compared to wild type (Fig. 4). The expression levels of the gene encoding GR in both transgenic lines TL-1 and TL-2 was five times higher than in wild type. The expression levels of the GST-encoding gene were 3.5 times higher in TL-1 and 2.8 times higher in TL-2. The expression level of the gene encoding APx was three times higher in both TL-1 and TL-2. The GPx-encoding gene showed 2.7 times higher expression levels in TL-1 than in wild type, and 2.4 times higher in TL-2 (Fig. 4).

### Influence of *AtROS1* overexpression on demethylase activity of transgenic tobacco

**Fig. 5.**
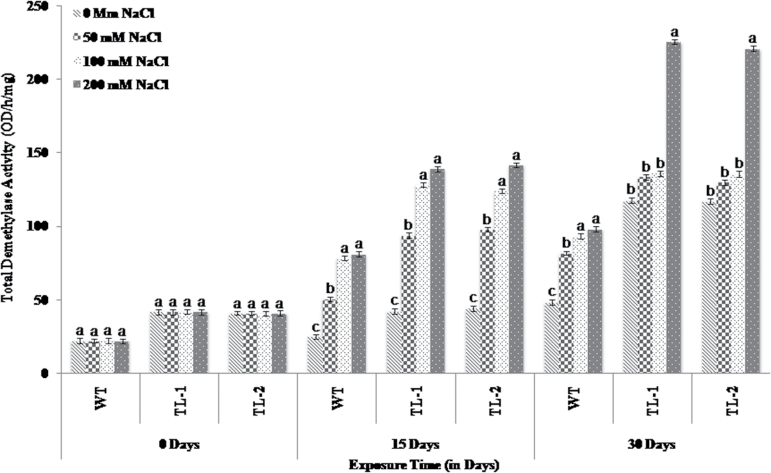
Total demethylase activity of wild-type and *AtROS1* transgenic tobacco lines under normal and salt stress conditions. Different alphabet on error bar represents significant difference. Different letters on error bar represents significant differences within treatment group, i.e. data marked ‘a’ is significantly different from data marked ‘b’ with *P* < 0.05, and from data marked ‘c’ with *P* < 0.01.

To evaluate the demethylase activity of transgenic lines vis-à-vis wild-type plants under control and salt stress conditions, nuclei were isolated and genome-wide total demethylase activity was analysed. *AtROS1* transgenic lines showed higher demethylase activity than wild-type plants under control conditions. In wild-type plants, total demethylase activity was enhanced from 22.06 OD/h/mg on day 0 to 48.25 OD/h/mg on day 30 under control conditions. In transgenic lines, total demethylase activity was increased from 41.74 OD/h/mg on day 0 to 117.55 OD/h/mg on day 30 for TL-1, and from 40.90 OD/h/mg on day 0 to 117 OD/h/mg on day 30 for TL-2 under control conditions. After 30 days, there was a greater increase in demethylase activity in transgenics relative to wild-type under control conditions (Fig. 5). Salt stress treatment was found to further enhance total demethylase activity of both wild-type and transgenic plants. During salt stress, total demethylase activity was increased from 22.06 OD/h/mg on day 0 to 98.04 OD/h/mg on day 30 in wild-type plants. In transgenic lines, total demethylase activity was dramatically increased from 41.74 OD/h/mg on day 0 to 225.65 OD/h/mg on day 30 in TL-1, and from 40.90 OD/h/mg on day 0 to 221.02 OD/h/mg on day 30 in TL-2 during salt stress (Fig. 5).

### Methylation mapping of promoter regions of the flavonoid biosynthetic and antioxidative systems through bisulfite sequencing

The methylation status of genes encoding enzymatic proteins of the flavonoid biosynthetic and antioxidative systems under normal versus salt stress conditions was performed by bisulfite sequencing of their promoter regions. Analysis of data revealed the methylation status of each cytosine present in amplified sequence in comparison to the reference sequence (Supplementary Fig. S2). In wild-type tobacco plant, three out of 14 cytosines in CHS, two out of eight cytosines in CHI, two out of seven cytosines in F3H, one out of five cytosines in FLS, and one out of six cytosines in DFR and ANS were found demethylated under control conditions. In *AtROS1* transgenic TL-1, five out of 14 cytosines in CHS, three out of eight cytosines in CHI, three out of seven cytosines in F3H, two out of five cytosines in FLS, two out of six cytosines in DFR, and two out of six cytosines in ANS were found demethylated. In *AtROS1* transgenic TL-2, four out of 14 cytosines in CHS, three out of eight cytosines in CHI, two out of seven cytosines in F3H, one out of five cytosines in FLS, one out of six cytosines in DFR, and two out of six cytosines in ANS were found demethylated under control conditions. Under salt stress condition, five out of 14 cytosines in CHS, three out of eight cytosines in CHI, three out of seven cytosines in F3H, two out of five cytosines in FLS, and three out of six cytosines in DFR and ANS were found demethylated in wild-type plants. In *AtROS1* transgenic TL-1, nine out of 14 cytosines in CHS, five out of eight cytosines in CHI, four out of seven cytosines in F3H, three out of five cytosines in FLS, and four out of six cytosines in DFR and ANS were found demethylated during salt stress conditions. In *AtROS1* transgenic TL-2, eight out of 14 cytosines in CHS, five out of eight cytosines in CHI, four out of seven cytosines in F3H, three out of five cytosines in FLS, and four out of six cytosines in DFR and ANS were found demethylated during salt stress conditions (Table 1).

**Table 1.** Tabular representation of demethylation status of CpGs found in the amplified segments of promoters of wild-type and transgenic lines under control and salt stress conditions after bisulfite sequencing

Target genes	Total number of CpGs	Demethylated CpGs					
		Control			200mM NaCl		
		WT	TL-1	TL-2	WT	TL-1	TL-2
CHS	14	3	5	4	5	9	8
CHI	8	2	3	3	3	5	5
F3H	7	2	3	2	3	4	4
FLS	5	1	2	1	2	3	3
DFR	6	1	2	1	3	4	4
ANS	6	1	2	2	3	4	4
GST	2	0	1	1	1	1	1
APx	6	1	2	2	2	3	3
GPx	4	1	2	2	2	3	3
GR	7	1	2	2	3	5	4

In promoters of antioxidative pathway genes, 0 out of two cytosines in GST, one out of six cytosines in APx, one out of four cytosines in GPx and one out of seven cytosines in GR were found demethylated in wild type under control conditions. In *AtROS1* transgenic TL-1, one out of two cytosines in GST, two out of six cytosines in APx, two out of four cytosines in GPx and two out of seven cytosines in GR were found demethylated under control conditions. In *AtROS1* transgenic TL-2, one out of two cytosines in GST, two out of six cytosines in APx, two out of four cytosines in GPx, and two out of seven cytosines in GR were found demethylated under control conditions. Under salt stress, one out of two cytosines in GST, two out of six cytosines in APx, two out of four cytosines in GPx, and three out of seven cytosines in GR were found demethylated in wild type. In *AtROS1* transgenic TL-1, one out of two cytosines in GST, three out of six cytosines in APx, three out of four cytosines in GPx, and five out of seven cytosines in GR were found demethylated under salt stress conditions. In *AtROS1* transgenic TL-2, one out of two cytosines in GST, three out of six cytosines in APx, three out of four cytosines in GPx, and four out of seven cytosines in GR were found demethylated under salt stress conditions (Table 1).

Taken together, *AtROS1* overexpression increased the number of demethylated cytosines in the promoter regions of genes encoding enzymes of the flavonoid biosynthetic and antioxidative systems under control conditions. Salt stress exposure of plants further increased the level of demethylated cytosines in wild-type as well as transgenic plants, suggesting the role of *AtROS1* in demethylation.

### Methylation status of coding regions of genes of the flavonoid biosynthetic and antioxidative systems

**Fig. 6.**
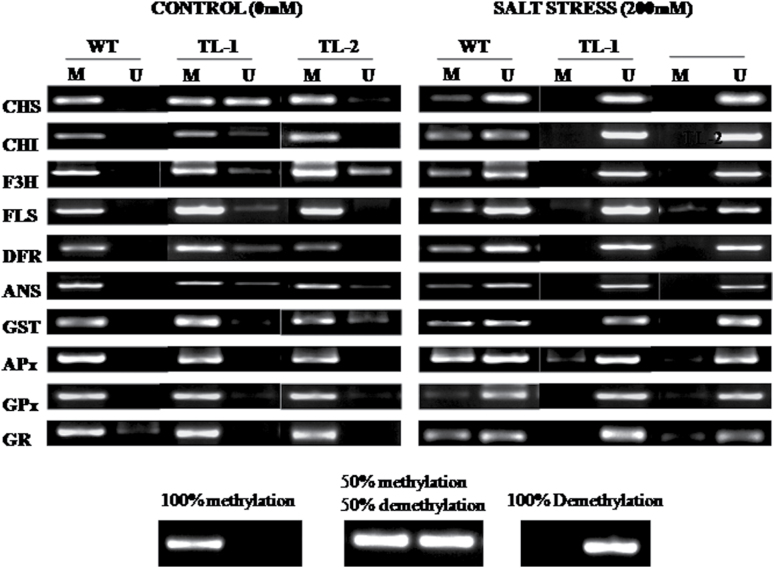
Methylation pattern of coding regions of genes encoding enzymes of the flavonoid and antioxidative pathways by methylation-specific PCR in wild-type and transgenic tobacco lines under normal and salt stress conditions. Methylation pattern of coding regions of genes encoding enzymes. M, methylated; U, unmethylated.

Analysis of the methylation status of the coding region of genes encoding enzymes of the flavonoid biosynthetic and antioxidative pathways was performed by methylation-specific PCR. Results showed significant differences in methylation frequencies of CpG sites of coding regions of genes of both these pathways. Under control conditions, amplification was observed only with primers designed for methylated coding regions in wild-type plants, which signifies that CpG sites of coding regions of genes of the flavonoid biosynthetic pathway were methylated. However, the appearance of a light band for GR under unmethylation along with an intense band in the methylation position suggest the presence of unmethylated CpG in GR as well. In transgenic lines, amplification was observed mostly for methylation of coding regions of genes of both the pathways under control conditions. However, light bands were observed for unmethylation for genes of the flavonoid biosynthetic pathway under control conditions, suggesting some unmethylated CpG sites in these genes. Similar to wild-type plants, CpGs in the coding regions of genes of the antioxidative pathway were mostly methylated in transgenic plants (Fig. 6).

Exposure of plants to salt stress was found to induce demethylation of CpGs in coding regions of genes of flavonoid biosynthetic and antioxidative pathways. In wild-type plants, both methylation and demethylation in CpGs of coding regions of various genes was observed under salt stress. The level of demethylation was higher compared to methylation in wild-type plants. In transgenic plants, amplification corresponding to demethylated CpG specific indicates that most of the CpGs of coding regions of genes of both the flavonoid biosynthetic and antioxidative pathway were demethylated under salt stress exposure (Fig. 6).

## Discussion

Crop production is adversely affected by abiotic stresses (Dolferus, 2014). At the advent of environmental risks, plants build up their defence system to protect themselves (Gill and Tuteja, 2010). But beyond certain limit, plants are not able to tolerate such adverse conditions with their natural defence systems. Thus it is important to develop plant varieties that show tolerance to stresses through subsequent generations. Studies have reported the influence of stresses on the expression levels of genes of the flavonoid biosynthesis and antioxidative systems (Badawi *et al.*, 2004; Ithal and Reddy, 2004; Tattini *et al.*, 2004; Lee *et al.*, 2007; Le Martret *et al.*, 2011). Salt stress has also been documented to affect the functioning of the flavonoid and antioxidative systems by influencing the regulation of genes encoding enzymes of these pathways at transcriptional as well as at post-transcriptional levels (Lee *et al.*, 2007; Yang *et al.*, 2009; Le Martret *et al.*, 2011). Here, what is believed to be the first study has been made of whether these pathways are under epigenetic regulation, particularly DNA methylation, during salt stress.

Epigenetic regulation involves DNA methylation and post-translational modification of histone proteins (Vaissière *et al.*, 2008). Changes in chromatin-associated factors are known to regulate the expression of stress responsive genes and plant development under stress (Chinnusamy and Zhu, 2009). Cytosine DNA methyltransferases are the main players in epigenetic regulation, through DNA methylation and modulation in expression levels of genes (Morales-Ruiz *et al.*, 2006). According to earlier reports, some stress-induced modifications are inherited by cell divisions as transgenerational stress memory (Chinnusamy and Zhu, 2009). ROS1/DME family DNA glycosylases perform an important role in plants by maintaining methylation patterns at the target sites within the genome (Zhu *et al.*, 2007; Roldán-Arjona and Ariza, 2009). The available data indicate that plant cells are able to repair damaged DNA as well as modified bases (5-meC) through a base excision repair pathway governed by the ROS1/DME family of DNA glycosylases (Roldán-Arjona and Ariza, 2009). A bifunctional DNA glycosylase/lyase ROS1 has been identified to be involved in DNA demethylation and to act as a repressor of transcriptional gene silencing by demethylation of cytosines, mainly at promoters of target genes (Gong *et al.*, 2002; Kapoor *et al.*, 2005; Agius *et al.*, 2006; Penterman *et al.*, 2007*a*; Penterman *et al.*, 2007*b*; Zhu *et al.*, 2007; Lister *et al.*, 2008; Zhu, 2009).

In view of this, transgenic tobacco plants overexpressing *AtROS1* were generated to study DNA methylation as an epigenetic regulation mark on the flavonoid biosynthetic and antioxidative pathways during salt stress. Environmental signals have been documented to reduce DNA methylation in *Arabidopsis* and *Zea mays* genome without DNA replication, providing evidence of stress-induced changes in methylation patterns of genes (Steward *et al.*, 2002; Pavet *et al.*, 2006). Demethylation largely activated the expression of genes and such changes are inherited through to the next generations (Akimoto *et al.*, 2007). To confirm this finding, wild-type and *AtROS1* overexpressing transgenic tobacco were analysed for the transcript expression of genes encoding enzymes of the flavonoid biosynthesis and antioxidative pathways during control and salt stress conditions. Exposure of plants to salt stress induced the expression of genes encoding enzymes of the flavonoid biosynthesis pathway (CHS, CHI, F3H, FLS, DFR, ANS) and the antioxidant pathway (GST, APx, GPx, GR). *AtROS1* transgenic tobacco plants showed greater induction in expression levels of these various genes compared to wild-type plants. *ros1 Arabidopsis* mutants have been reported for hypermethylation of genes/transgenes leading to transcriptional gene silencing (Gong *et al.*, 2002). On the contrary, ROS1 represses transcriptional gene silencing by demethylation leading to the activation of gene expressions. Hence, the observed higher levels of expression of various genes during stress condition in *AtROS1* transgenic tobacco could be correlated with demethylase activity.

Because ROS1 governs the demethylation, total demethylase activity of *AtROS1* transgenic tobacco plants was increased upon exposure to salt stress. In RNA-dependent DNA methylation mutants, the mRNA level of ROS1 is very low because, in response to environmental disturbances, the accretion pattern of ROS1 protein may vary with the plant genome’s methylation status (Huettel *et al.*, 2006; Mathieu *et al.*, 2007; Zhu, 2009). A greater increase in demethylase activity of *AtROS1* transgenic tobacco during salt stress compared to wild-type plants indicates the significance of *AtROS1* overexpression or the role of ROS1 in DNA demethylation.

In mammals, CpG islands are epigenetically regulated by DNA methylation (Carninci *et al.*, 2006). In *Oryza sativa*, similar methylation of CpG islands has been reported in the promoter regions of genes that reduced their expression levels (Akimoto *et al.*, 2007). Thus, to see whether demethylation occurred in the promoters and/or coding regions of genes encoding enzymes of the flavonoid biosynthesis and antioxidative pathways, methylation status was mapped by bisulfite sequencing of the promoters and by methylation-specific PCR of the coding regions of genes. ROS1 and DME gave preference to symmetric CpG over CpNpG and asymmetric sequences for their demethylase activity (Morales-Ruiz *et al.*, 2006). There were more demethylated CpGs in promoters of genes encoding enzymes of the flavonoid biosynthesis and antioxidant pathways in *AtROS1* transgenic tobacco compared to wild-type under control conditions. The level of demethylation in the promoter regions was further increased in *AtROS1* transgenic tobacco compared to wild-type during salt stress conditions. An *in vitro* analysis of ROS1 protein activity on methylated plasmid DNA has suggested that is has a role in nick formation in a sequence-specific manner (Gong *et al.*, 2002). The data presented here suggest the role of *AtROS1* in the demethylation of promoters of genes encoding enzymes of the flavonoid biosynthesis and antioxidative pathways during control and salt stress conditions.

DNA methylation is a conserved system and methylation of gene body/coding regions of a gene is an evolutionary characteristic of eukaryotic genomes (Zemach *et al.*, 2010; Takuno and Gaut, 2012). A recent study has found that methylation within the gene body plays an important role in regulating the expression of stress-related genes (Karan *et al.*, 2012). Methylation near the 5′ end of coding region has been reported to be associated with gene silencing (Hohn *et al.*, 1996; Zilberman *et al.*, 2007; To and Kim, 2014). Gene body methylation may occur to prevent aberrant expression from intragenic promoters or to enhance the accuracy of splicing (Maunakea *et al.*, 2010; You *et al.*, 2012). Methylation-specific PCR revealed the methylation status for coding regions of genes encoding enzymes of the flavonoid biosynthetic and antioxidative pathways in *AtROS1* transgenic lines compared to wild-type plants under control and salt stress conditions. In tobacco plants, coding regions of *NtGPDL*-like genes have been reported to rapidly undergo demethylation upon abiotic stress exposures (Choi and Sano, 2007). Here, in wild-type tobacco, salt stress induced significant levels of demethylation in coding regions of genes encoding enzymes of the flavonoid biosynthesis and antioxidative pathways (CHS, CHI, F3H, FLS, DFR, ANS, GR, GST, APx, and GPx). By comparison, these coding regions were completely demethylated in *AtROS1* transgenic lines under salt stress conditions.

In conclusion, this study documents for the first time that overexpression of *AtROS1* demethylates the promoter as well as the coding regions of genes encoding enzymes of the flavonoid biosynthesis and antioxidative pathways. This could be the reason for the observed improvement in salt stress tolerance of *AtROS1* transgenic tobacco compared to wild-type tobacco plants.

## Supplementary data

Supplementary material is available at *JXB* online.

Supplementary Fig. S1. Generation of *AtROS1* overexpressing transgenic tobacco.

Supplementary Fig. S2. Sequence alignment of bisulfite converted and unconverted DNA showing the methylation marks in the promoter regions of genes encoding enzymatic proteins of flavonoid biosynthetic and antioxidative pathways.

Supplementary Table S1. Oligonucleotide sequences used for identification of *AtROS1* overexpressing transgenic tobacco.

Supplementary Table S2. Oligonucleotide sequences used in relative transcript expression analysis of genes by quantitative real-time PCR.

Supplementary Table S3. Oligonucleotide sequences used for genome walking.

Supplementary Table S4. Oligonucleotide sequences designed for bisulfite sequencing of promoters of genes encoding enzymes of the flavonoid biosynthesis and antioxidative pathways.

Supplementary Table S5. Oligonucleotide sequences for methylation specific PCR corresponding to methylated region of coding sequences of genes encoding enzymes of the flavonoid biosynthesis and antioxidative pathways.

Supplementary Table S6. Oligonucleotide sequences for methylation-specific PCR corresponding to unmethylated region of coding sequences of genes encoding enzymes of the flavonoid biosynthesis and antioxidative pathways.

Supplementary Data

## References

[CIT0001] Agius F Kapoor A Zhu J-K 2006 Role of the *Arabidopsis* DNA glycosylase/lyase ROS1 in active DNA demethylation. Proceedings of the National Academy of Sciences of the United States of America 103, 11796–11801. 16864782 10.1073/pnas.0603563103 PMC1544249

[CIT0002] Akimoto K Katakami H Kim H-J Ogawa E Sano CM Wada Y Sano H 2007 Epigenetic inheritance in rice plants. Annals of Botany 100, 205–217. 17576658 10.1093/aob/mcm110 PMC2735323

[CIT0003] Ashraf M 2009 Biotechnological approach of improving plant salt tolerance using antioxidants as markers. Biotechnology Advances 27, 84–93. 18950697 10.1016/j.biotechadv.2008.09.003

[CIT0004] Badawi GH Kawano N Yamauchi Y Shimada E Sasaki R Kubo A Tanaka K 2004 Over-expression of ascorbate peroxidase in tobacco chloroplasts enhances the tolerance to salt stress and water deficit. Physiologia Plantarum 121, 231–238. 15153190 10.1111/j.0031-9317.2004.00308.x

[CIT0005] Bird A 2002 DNA methylation patterns and epigenetic memory. Genes & Development 16, 6–21. 11782440 10.1101/gad.947102

[CIT0006] Bock C Reither S Mikeska T Paulsen M Walter J Lengauer T 2005 BiQ Analyzer: Visualization and quality control for DNA methylation data from bisulfite sequencing. Bioinformatics 21, 4067–4068. 16141249 10.1093/bioinformatics/bti652

[CIT0007] Bors W Heller W Michel C Saran M 1990 Flavonoids as antioxidants: determination of radical-scavenging efficiencies. Methods in Enzymology 186, 343–355. 2172711 10.1016/0076-6879(90)86128-i

[CIT0008] Carninci P Sandelin A Lenhard B 2006 Genome-wide analysis of mammalian promoter architecture and evolution. Nature Genetics 38, 626–635. 16645617 10.1038/ng1789

[CIT0009] Chinnusamy V Zhu J-K 2009 Epigenetic regulation of stress responses in plants. Current Opinion in Plant Biology 12, 133–139. 19179104 10.1016/j.pbi.2008.12.006 PMC3139470

[CIT0010] Choi Y Gehring M Johnson L Hannon M Harada JJ Goldberg RB Jacobsen SE Fischer RL 2002 DEMETER, a DNA glycosylase domain protein, is required for endosperm gene imprinting and seed viability in *Arabidopsis* . Cell 110, 33–42. 12150995 10.1016/s0092-8674(02)00807-3

[CIT0011] Choi CS Sano H 2007 Abiotic-stress induces demethylation and transcriptional activation of a gene encoding a glycerophosphodiesterase-like protein in tobacco plants. Molecular Genetics and Genomics 277, 589–600. 17273870 10.1007/s00438-007-0209-1

[CIT0012] Dolferus R 2014 To grow or not to grow: a stressful decision for plants. Plant Science 229C, 247–261. 25443851 10.1016/j.plantsci.2014.10.002

[CIT0013] Fini A Brunetti C Di Ferdinando M Ferrini F Tattini M 2011 Stress-induced flavonoid biosynthesis and the antioxidant machinery of plants. Plant Signaling & Behavior 6, 709–711. 21448007 10.4161/psb.6.5.15069 PMC3172844

[CIT0014] Finnegan EJ Kovac KA 2000 Plant DNA methyltransferases. Plant Molecular Biology 43, 189–201. 10999404 10.1023/a:1006427226972

[CIT0015] Furner IJ Matzke M 2011 Methylation and demethylation of the *Arabidopsis* genome. Current Opinion in Plant Biology 14, 137–141. 21159546 10.1016/j.pbi.2010.11.004

[CIT0016] Gehring M Huh JH Hsieh TF Penterman J Choi Y Harada JJ Goldberg RB Fischer RL 2006 DEMETER DNA glycosylase establishes MEDEA polycomb gene self-imprinting by allele-specific demethylation. Cell 124, 495–506. 16469697 10.1016/j.cell.2005.12.034 PMC4106368

[CIT0017] Ghasemzadeh A Ghasemzadeh N 2011 Flavonoids and phenolic acids: role and biochemical activity in plants and human. Journal of Medicinal Plants Research 5, 6697–6703.

[CIT0018] Ghawana S Paul A Kumar H 2011 An RNA isolation system for plant tissues rich in secondary metabolites. BMC Research Notes 4, 85. 21443767 10.1186/1756-0500-4-85 PMC3079660

[CIT0019] Gill SS Tuteja N 2010 Reactive oxygen species and antioxidant machinery in abiotic stress tolerance in crop plants. Plant Physiology and Biochemistry: PPB / Société Française de Physiologie Végétale **48**, 909–930. 10.1016/j.plaphy.2010.08.016 20870416

[CIT0020] Gong Z Morales-Ruiz T Ariza RR Roldán-Arjona T David L Zhu JK 2002 ROS1, a repressor of transcriptional gene silencing in *Arabidopsis*, encodes a DNA glycosylase/lyase. Cell 111, 803–814. 12526807 10.1016/s0092-8674(02)01133-9

[CIT0021] Greenway H Munns R 1980 Mechanisms of salt tolerance in nonhalophytes. Annual Review of Plant Physiology 31, 149–190.

[CIT0022] Gupta B Huang B 2014 Mechanism of salinity tolerance in plants: physiological, biochemical, and molecular characterization. International Journal of Genomics 2014, 18. 10.1155/2014/701596 PMC3996477 24804192

[CIT0023] Hauser MT Aufsatz W Jonak C Luschnig C 2011 Transgenerational epigenetic inheritance in plants. Biochimica et Biophysica Acta - Gene Regulatory Mechanisms 1809, 459–468. 10.1016/j.bbagrm.2011.03.007 PMC4359895 21515434

[CIT0024] Henderson IR Jacobsen SE 2007 Epigenetic inheritance in plants. Nature 447, 418–424. 17522675 10.1038/nature05917

[CIT0025] Herman JG Graff JR Myöhänen S Nelkin BD Baylin SB 1996 Methylation-specific PCR: a novel PCR assay for methylation status of CpG islands. Proceedings of the National Academy of Sciences of the United States of America 93, 9821–9826. 8790415 10.1073/pnas.93.18.9821 PMC38513

[CIT0026] Hohn T Corsten S Rieke S Müller M Rothnie H 1996 Methylation of coding region alone inhibits gene expression in plant protoplasts. Proceedings of the National Academy of Sciences of the United States of America 93, 8334–8339. 8710871 10.1073/pnas.93.16.8334 PMC38671

[CIT0027] Huettel B Kanno T Daxinger L Aufsatz W Matzke AJM Matzke M 2006 Endogenous targets of RNA-directed DNA methylation and Pol IV in *Arabidopsis* . The EMBO Journal 25, 2828–2836. 16724114 10.1038/sj.emboj.7601150 PMC1500864

[CIT0028] Ithal N Reddy AR 2004 Rice flavonoid pathway genes, OsDfr and OsAns, are induced by dehydration, high salt and ABA, and contain stress responsive promoter elements that interact with the transcription activator, OsC1-MYB. Plant Science 166, 1505–1513.

[CIT0029] Jouyban Z 2012 The effects of salt stress on plant growth. Technical Journal of Engineering and Applied Sciences 2, 7–10.

[CIT0030] Kapoor A Agius F Zhu JK 2005 Preventing transcriptional gene silencing by active DNA demethylation. FEBS Letters 579, 5889–5898. 16162337 10.1016/j.febslet.2005.08.039

[CIT0031] Karan R DeLeon T Biradar H Subudhi PK 2012 Salt stress induced variation in DNA methylation pattern and its influence on gene expression in contrasting rice genotypes. PLoS ONE 7, e40203. 22761959 10.1371/journal.pone.0040203 PMC3386172

[CIT0032] Krokan HE Standal R Slupphaug G 1997 DNA glycosylases in the base excision repair of DNA. Biochemistry Journal 325, 1–16. 10.1042/bj3250001 PMC1218522 9224623

[CIT0033] Lee YP Kim SH Bang JW Lee HS Kwak SS Kwon SY 2007 Enhanced tolerance to oxidative stress in transgenic tobacco plants expressing three antioxidant enzymes in chloroplasts. Plant Cell Reports 26, 591–598. 17268803 10.1007/s00299-006-0253-z

[CIT0034] Li L-C Dahiya R 2002 MethPrimer: designing primers for methylation PCRs. Bioinformatics (Oxford, England) 18, 1427–1431. 10.1093/bioinformatics/18.11.1427 12424112

[CIT0035] Li G Peng X Wei L Kang G 2013 Salicylic acid increases the contents of glutathione and ascorbate and temporally regulates the related gene expression in salt-stressed wheat seedlings. Gene 529, 321–325. 23948081 10.1016/j.gene.2013.07.093

[CIT0036] Li X Hou S Gao Q Zhao P Chen S Qi D Lee BH Cheng L Liu G 2013 LcSAIN1, a novel salt-induced gene from sheepgrass, confers salt stress tolerance in transgenic *Arabidopsis* and rice. Plant and Cell Physiology 54, 1172–1185. 23695503 10.1093/pcp/pct069

[CIT0037] Lister R O’Malley RC Tonti-Filippini J Gregory BD Berry CC Millar AH Ecker JR 2008 Highly integrated single-base resolution maps of the epigenome in *Arabidopsis* . Cell 133, 523–536. 18423832 10.1016/j.cell.2008.03.029 PMC2723732

[CIT0038] Le Martret B Poage M Shiel K Nugent GD Dix PJ 2011 Tobacco chloroplast transformants expressing genes encoding dehydroascorbate reductase, glutathione reductase, and glutathione-s-transferase, exhibit altered anti-oxidant metabolism and improved abiotic stress tolerance. Plant Biotechnology Journal 9, 661–673. 21450042 10.1111/j.1467-7652.2011.00611.x

[CIT0039] Mathieu O Reinders J Čaikovski M Smathajitt C Paszkowski J 2007 Transgenerational stability of the *Arabidopsis* epigenome is coordinated by CG methylation. Cell 130, 851–862. 17803908 10.1016/j.cell.2007.07.007

[CIT0040] Matus JT Poupin MJ Cañón P Bordeu E Alcalde JA Arce-Johnson P 2010 Isolation of WDR and bHLH genes related to flavonoid synthesis in grapevine (*Vitis vinifera* L.). Plant Molecular Biology 72, 607–620. 20112051 10.1007/s11103-010-9597-4

[CIT0041] Maunakea AK Nagarajan RP Bilenky M 2010 Conserved role of intragenic DNA methylation in regulating alternative promoters. Nature 466, 253–257. 20613842 10.1038/nature09165 PMC3998662

[CIT0042] Morales-Ruiz T Ortega-Galisteo AP Ponferrada-Marín MI Martínez-Macías MI Ariza RR Roldán-Arjona T 2006 DEMETER and REPRESSOR OF SILENCING 1 encode 5-methylcytosine DNA glycosylases. Proceedings of the National Academy of Sciences of the United States of America 103, 6853–6858. 16624880 10.1073/pnas.0601109103 PMC1458983

[CIT0043] Pavet V Quintero C Cecchini NM Rosa AL Alvarez ME 2006 *Arabidopsis* displays centromeric DNA hypomethylation and cytological alterations of heterochromatin upon attack by pseudomonas syringae. Molecular Plant-Microbe Interactions: MPMI 19, 577–587. 10.1094/MPMI-19-0577 16776291

[CIT0044] Penterman J Zilberman D Huh JH Ballinger T Henikoff S Fischer RL 2007 *a* DNA demethylation in the *Arabidopsis* genome. Proceedings of the National Academy of Sciences of the United States of America 104, 6752–6757. 17409185 10.1073/pnas.0701861104 PMC1847597

[CIT0045] Penterman J Uzawa R Fischer RL 2007 *b* Genetic interactions between DNA demethylation and methylation in *Arabidopsis* . Plant Physiology 145, 1549–1557. 17951456 10.1104/pp.107.107730 PMC2151691

[CIT0046] Ponferrada-Marín MI Martínez-Macías MI Morales-Ruiz T Roldán-Arjona T Ariza RR 2010 Methylation-independent DNA binding modulates specificity of repressor of silencing 1 (ROS1) and facilitates demethylation in long substrates. Journal of Biological Chemistry 285, 23032–23039. 20489198 10.1074/jbc.M110.124578 PMC2906296

[CIT0047] Ponferrada-Marín MI Roldán-Arjona T Ariza RR 2009 ROS1 5-methylcytosine DNA glycosylase is a slow-turnover catalyst that initiates DNA demethylation in a distributive fashion. Nucleic Acids Research 37, 4264–4274. 19443451 10.1093/nar/gkp390 PMC2715244

[CIT0048] Ponferrada-Marín MI Roldán-Arjona T Ariza RR 2012 Demethylation initiated by ROS1 glycosylase involves random sliding along DNA. Nucleic Acids Research 40, 11554–11562. 23034804 10.1093/nar/gks894 PMC3526269

[CIT0049] Rodriguez Milla MA Maurer A Huete AR Gustafson JP 2003 Glutathione peroxidase genes in *Arabidopsis* are ubiquitous and regulated by abiotic stresses through diverse signaling pathways. Plant Journal 36, 602–615. 14617062 10.1046/j.1365-313x.2003.01901.x

[CIT0050] Roldán-Arjona T Ariza RR 2009 DNA demethylation. Madame Curie Bioscience Database [Internet]. Austin, TX: Landes Bioscience; 2000. Available from: http://www.ncbi.nlm.nih.gov/books/NBK6365/ 157–169.

[CIT0051] Roxas VP Lodhi SA Garrett DK Mahan JR Allen RD 2000 Stress tolerance in transgenic tobacco seedlings that overexpress glutathione S-transferase/glutathione peroxidase. Plant & Cell Physiology 41, 1229–1234. 11092907 10.1093/pcp/pcd051

[CIT0052] Sairam RK Rao KV Srivastava GC 2002 Differential response of wheat genotypes to long term salinity stress in relation to oxidative stress, antioxidant activity and osmolyte concentration. Plant Science 163, 1037–1046.

[CIT0053] Sharma P Jha AB Dubey RS Pessarakli M 2012 Reactive oxygen species, oxidative damage, and antioxidative defense mechanism in plants under stressful conditions. Journal of Botany 2012, 1–26.

[CIT0054] Shinozaki K Yamaguchi-Shinozaki K 1997 Gene expression and signal transduction in water-stress response. Plant Physiology 115, 327–334. 12223810 10.1104/pp.115.2.327 PMC158490

[CIT0055] Singh DP Prabha R Meena KK Sharma L Sharma AK 2014 Induced accumulation of polyphenolics and flavonoids in cyanobacteria under salt stress protects organisms through enhanced antioxidant activity. American Journal of Plant Sciences 05, 726–735.

[CIT0056] Steward N Ito M Yamaguchi Y Koizumi N Sano H 2002 Periodic DNA methylation in maize nucleosomes and demethylation by environmental stress. Journal of Biological Chemistry 277, 37741–37746. 12124387 10.1074/jbc.M204050200

[CIT0057] Takuno S Gaut BS 2012 Body-methylated genes in *Arabidopsis thaliana* are functionally important and evolve slowly. Molecular Biology and Evolution 29, 219–227. 21813466 10.1093/molbev/msr188

[CIT0058] Tattini M Galardi C Pinelli P Massai R Remorini D Agati G 2004 Differential accumulation of flavonoids and hydroxycinnamates in leaves of *Ligustrum vulgare* under excess light and drought stress. New Phytologist 163, 547–561. 10.1111/j.1469-8137.2004.01126.x 33873733

[CIT0059] To TK Kim JM 2014 Epigenetic regulation of gene responsiveness in *Arabidopsis* . Frontiers in Plant Science 4, 548. 24432027 10.3389/fpls.2013.00548 PMC3882666

[CIT0060] Vaissière T Sawan C Herceg Z 2008 Epigenetic interplay between histone modifications and DNA methylation in gene silencing. Mutation Research - Reviews in Mutation Research 659, 40–48. 18407786 10.1016/j.mrrev.2008.02.004

[CIT0061] Walia H Wilson C Condamine P 2005 Comparative transcriptional profiling of two contrasting rice genotypes under salinity stress during the vegetative growth stage. Plant Physiology 139, 822–835. 16183841 10.1104/pp.105.065961 PMC1255998

[CIT0062] Xiong L Schumaker KS Zhu J-K 2002 Cell signaling during cold, drought, and salt stress. The Plant Cell 14 Suppl, S165–S183. 12045276 10.1105/tpc.000596 PMC151254

[CIT0063] Yang Q Chen ZZ Zhou XF Yin HB Li X Xin XF Hong XH Zhu JK Gong Z 2009 Overexpression of SOS (salt overly sensitive) genes increases salt tolerance in transgenic *Arabidopsis* . Molecular Plant 2, 22–31. 19529826 10.1093/mp/ssn058 PMC2639737

[CIT0064] You W Tyczewska A Spencer M Daxinger L Schmid MW Grossniklaus U Simon SA Meyers BC Matzke AJ Matzke M 2012 Atypical DNA methylation of genes encoding cysteine-rich peptides in *Arabidopsis thaliana* . BMC Plant Biology 12, 51. 22512782 10.1186/1471-2229-12-51 PMC3422182

[CIT0065] Zemach A McDaniel IE Silva P Zilberman D 2010 Genome-wide evolutionary analysis of eukaryotic DNA methylation. Science (New York, NY) 328, 916–919. 10.1126/science.1186366 20395474

[CIT0066] Zhu J-K 2009 Active DNA demethylation mediated by DNA glycosylases. Annual Review of Genetics 43, 143–166. 10.1146/annurev-genet-102108-134205 PMC3137514 19659441

[CIT0067] Zhu J Kapoor A Sridhar V V. Agius F Zhu JK 2007 The DNA Glycosylase/Lyase ROS1 functions in pruning DNA methylation patterns in *Arabidopsis* . Current Biology 17, 54–59. 17208187 10.1016/j.cub.2006.10.059

[CIT0068] Zilberman D Gehring M Tran RK Ballinger T Henikoff S 2007 Genome-wide analysis of *Arabidopsis thaliana* DNA methylation uncovers an interdependence between methylation and transcription. Nature Genetics 39, 61–69. 17128275 10.1038/ng1929

