# Supplementary material for: AtROS1 overexpression provides evidence for epigenetic regulation of genes encoding enzymes of flavonoid biosynthesis and antioxidant pathways during salt stress in transgenic tobacco
Source: J Exp Bot. 2015 Jun 25;66(19):5959–69. doi: 10.1093/jxb/erv304 (PMC4566984; doi:10.1093/jxb/erv304)
Supplement: Supplementary Data [file supp_66_19_5959__index.html]

AtROS1 overexpression provides evidence for epigenetic regulation of genes encoding enzymes of flavonoid biosynthesis and antioxidant pathways during salt stress in transgenic tobacco — AtROS1 overexpression provides evidence for epigenetic regulation of genes encoding enzymes of flavonoid biosynthesis and antioxidant pathways during salt stress in transgenic tobacco — Supplementary Data 

# AtROS1 overexpression provides evidence for epigenetic regulation of genes encoding enzymes of flavonoid biosynthesis and antioxidant pathways during salt stress in transgenic tobacco

## Supplementary Data

Data files

- Supplementary Data - Supplementary Data
