# Supplementary material for: AtROS1 overexpression provides evidence for epigenetic regulation of genes encoding enzymes of flavonoid biosynthesis and antioxidant pathways during salt stress in transgenic tobacco
Source: J Exp Bot. 2015 Jun 25;66(19):5959–69. doi: 10.1093/jxb/erv304 (PMC4566984; doi:10.1093/jxb/erv304)
Supplement: Supplementary Data [file supp_erv304_jexbot147090_file001.pdf]

Supplementary data

AtROS1 overexpression provided evidence for epigenetic regulation of genes encoding enzymes of flavonoid biosynthesis and antioxidant pathways during salt stress in transgenic tobacco

Poonam Bharti, Monika Mahajan, Ajay K. Vishwakarma, Jyoti Bhardwaj and Sudesh Kumar Yadav*

Biotechnology Division, CSIR-Institute of Himalayan Bioresource Technology, Palampur 176061, Himanchal Pradesh, India.

* To whom correspondence should be addressed. E-mail skyt@rediffmail.com; sudeshkumar@ihbt.res.in

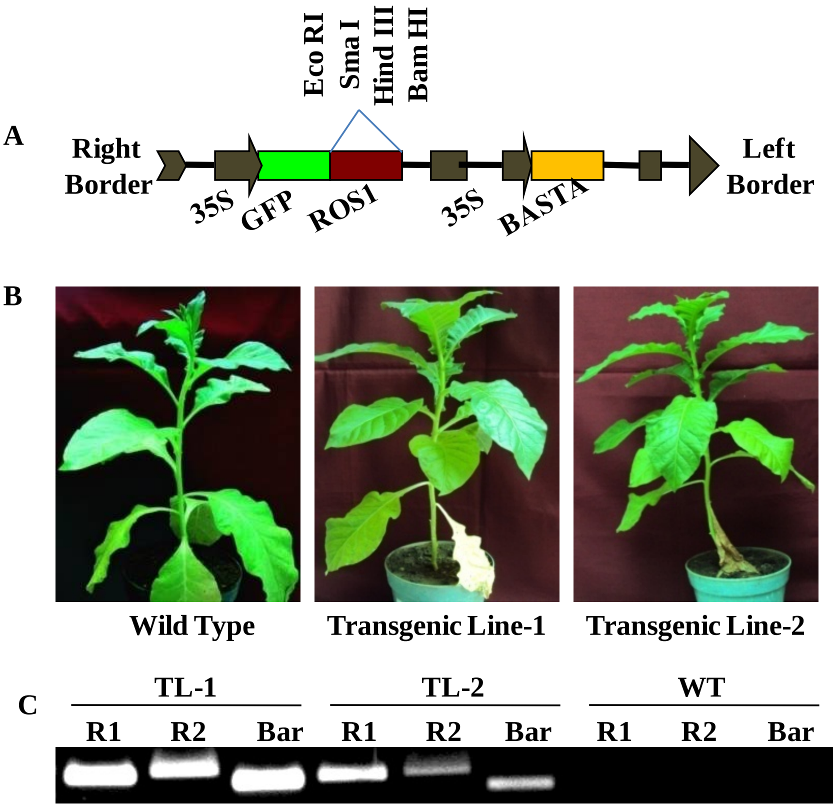

Figure S1. Generation of *AtROS1* overexpressing transgenic tobacco. (A) Schematic diagram of pEGAD-ROS1 expression construct used for tobacco transformation. (B) *AtROS1* overexpressing transgenic lines and control wild type tobacco plants. (C) Identification of transgenic lines by genomic DNA PCR analysis with gene specific primers. **R1**: Internal Primer 1, **R2**: Internal Primer 2 and **Bar**: BASTA selection marker specific primer, **WT**: wild type tobacco plants, **TL-1** and **TL-2**: transgenic tobacco lines.

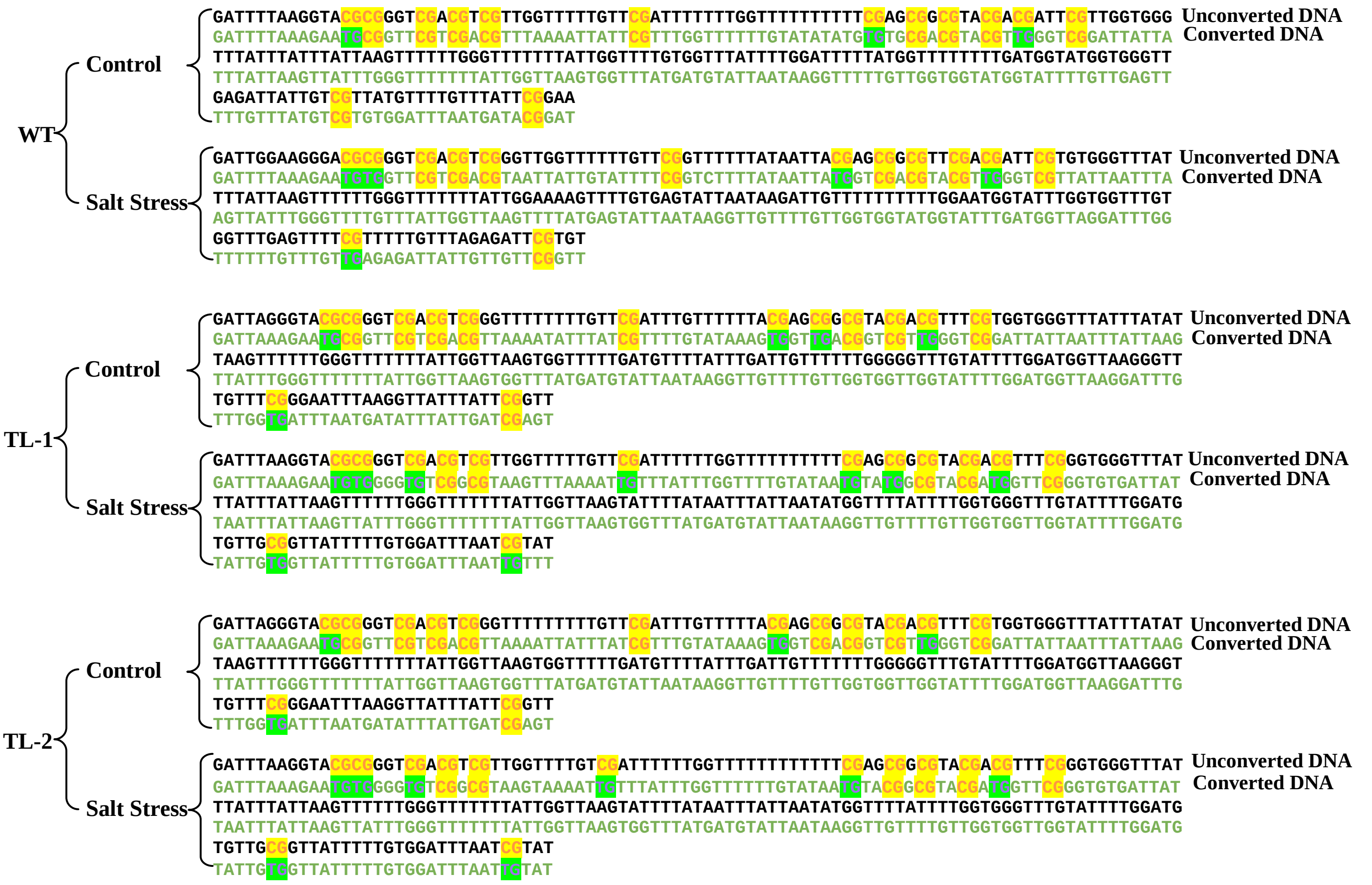
WT
Control
Salt Stress
TL-1
Control
Salt Stress
TL-2
Control
Salt Stress
Unconverted DNA
Converted DNA

(1)

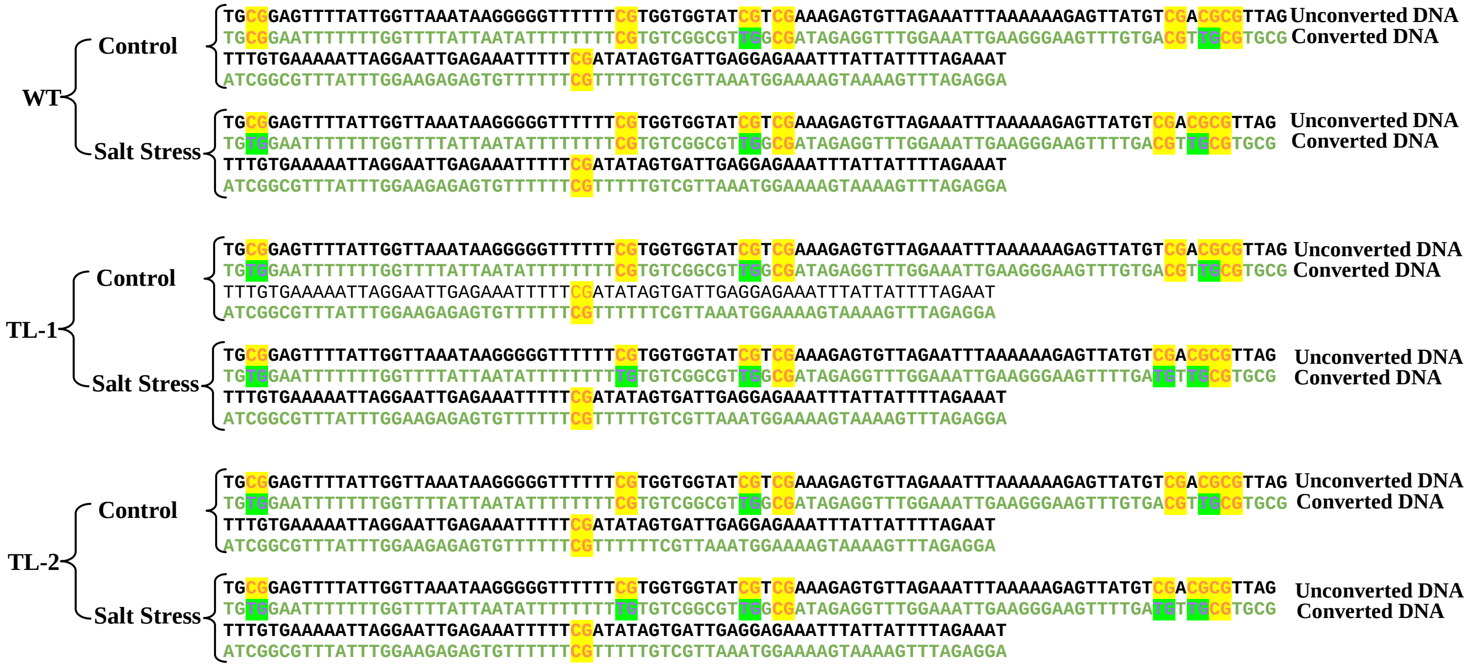
WT
Control
Salt Stress
TL-1
Control
Salt Stress
TL-2
Control
Salt Stress
Unconverted DNA
Converted DNA

(2)

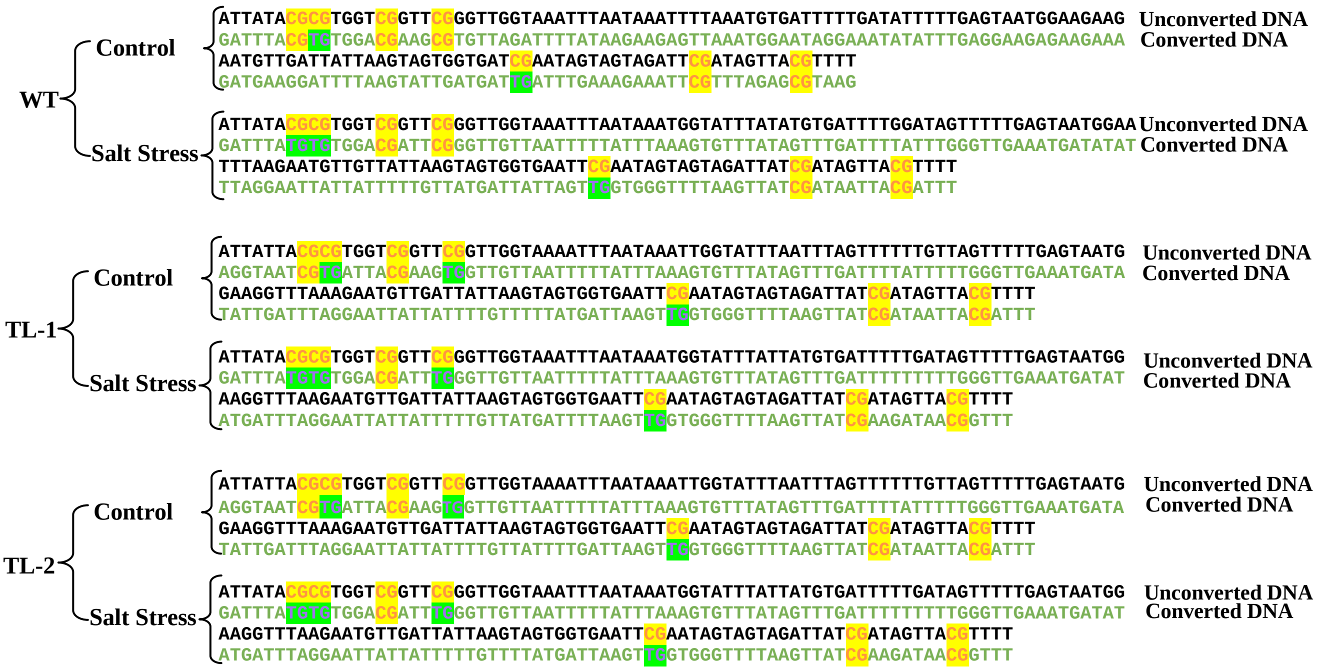
WT
Control
ATTATACGCGTGGTCGGTTCGGGTTGGTAAATTTAATAAATTTTAAATGTGATTTTTGATATTTTTGAGTAATGGAAGAAG Unconverted DNA
GATTTACGTGTGGACGAAGCGTGTTAGATTTTATAAGAAGAGTTAAATGGAATAGGAAATATATTTGAGGAAGAGAAGAAA Converted DNA
AATGTTGATTATTAAGTAGTGGTGATCGAATAGTAGTAGATTCGATAGTTACGTTTT
GATGAAGGATTTTAAGTATTGATGATTGATTTGAAAGAAATTCGTTTAGAGCGTAAG
Salt Stress
ATTATACGCGTGGTCGGTTCGGGTTGGTAAATTTAATAAATGGTATTTATATGTGATTTTGGATAGTTTTTGAGTAATGGAA Unconverted DNA
GATTTATGTGTGGACGATTCGGGTTGTTAATTTTTATTTAAAGTGTTTATAGTTTGATTTTATTTGGGTTGAAATGATATAT Converted DNA
TTTAAGAATGTTGTTATTAAGTAGTGGTGAATTCGAATAGTAGTAGATTATCGATAGTTACGTTTT
TTAGGAATTATTATTTTTGTTATGATTATTAGTTGGTGGGTTTTAAGTTATCGATAATTACGATTT
TL-1
Control
ATTATTACGCGTGGTCGGTTCGGTTGGTAAAATTTAATAAATTGGTATTTAATTTAGTTTTTTGTTAGTTTTTGAGTAATG Unconverted DNA
AGGTAATCGTGATTACGAAGTGGTTGTTAATTTTTATTTAAAGTGTTTATAGTTTGATTTTATTTTTGGGTTGAAATGATA Converted DNA
GAAGGTTTAAAGAATGTTGATTATTAAGTAGTGGTGAATTCGAATAGTAGTAGATTATCGATAGTTACGTTTT
TATTGATTTAGGAATTATTATTTTGTTTTTATGATTAAGTTGGTGGGTTTTAAGTTATCGATAATTACGATTT
Salt Stress
ATTATACGCGTGGTCGGTTCGGGTTGGTAAATTTAATAAATGGTATTTATTATGTGATTTTTGATAGTTTTTGAGTAATGG Unconverted DNA
GATTTATGTGTGGACGATTTGGGTTGTTAATTTTTATTTAAAGTGTTTATAGTTTGATTTTTTTTTGGGTTGAAATGATAT Converted DNA
AAGGTTTAAGAATGTTGATTATTAAGTAGTGGTGAATTCGAATAGTAGTAGATTATCGATAGTTACGTTTT
ATGATTTAGGAATTATTATTTTTGTTATGATTTTAAGTTGGTGGGTTTTAAGTTATCGAAGATAACGGTTT
TL-2
Control
ATTATTACGCGTGGTCGGTTCGGTTGGTAAAATTTAATAAATTGGTATTTAATTTAGTTTTTTGTTAGTTTTTGAGTAATG Unconverted DNA
AGGTAATCGTGATTACGAAGTGGTTGTTAATTTTTATTTAAAGTGTTTATAGTTTGATTTTATTTTTGGGTTGAAATGATA Converted DNA
GAAGGTTTAAAGAATGTTGATTATTAAGTAGTGGTGAATTCGAATAGTAGTAGATTATCGATAGTTACGTTTT
TATTGATTTAGGAATTATTATTTTGTTATTTTGATTAAGTTGGTGGGTTTTAAGTTATCGATAATTACGATTT
Salt Stress
ATTATACGCGTGGTCGGTTCGGGTTGGTAAATTTAATAAATGGTATTTATTATGTGATTTTTGATAGTTTTTGAGTAATGG Unconverted DNA
GATTTATGTGTGGACGATTTGGGTTGTTAATTTTTATTTAAAGTGTTTATAGTTTGATTTTTTTTTGGGTTGAAATGATAT Converted DNA
AAGGTTTAAGAATGTTGATTATTAAGTAGTGGTGAATTCGAATAGTAGTAGATTATCGATAGTTACGTTTT
ATGATTTAGGAATTATTATTTTTGTTTTATGATTAAGTTGGTGGGTTTTAAGTTATCGAAGATAACGGTTT

(3)

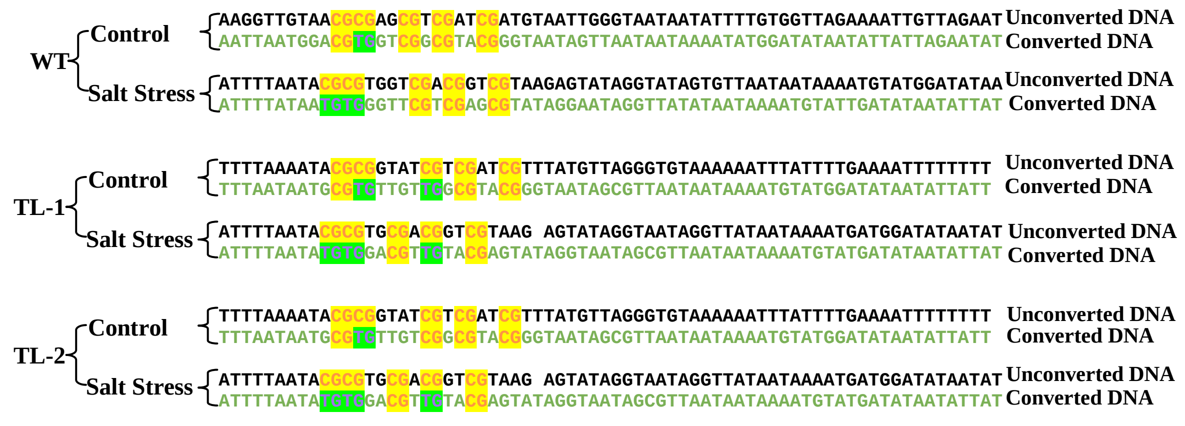
WT
Control
AAGGTTGTAACGCGAGCGTCGATCGATGTAATTGGGTAATAATATTTTGTGGTTAGAAAATTGTTAGAAT Unconverted DNA
AATTAATGGACGTGGTCGGCGTACGGGTAATAGTTAATAATAAAATATGGATATAATATTATTAGAATAT Converted DNA
Salt Stress
ATTTTAATACGCGTGGTCGACGGTCGTAAGAGTATAGGTATAGTGTTAATAATAAAATGTATGGATATAA Unconverted DNA
ATTTTATAATGTGGGTTCGTCGAGCGTATAGGAATAGGTTATATAATAAAATGTATTGATATAATATTAT Converted DNA
TL-1
Control
TTTTAAAATACGCGGTATCGTCGATCGTTTATGTTAGGGTGTAAAAAATTTATTTTGAAAATTTTTTTT Unconverted DNA
TTTAATAATGCGTGTTGTTGGCGTACGGGTAATAGCGTTAATAATAAAATGTATGGATATAATATTATT Converted DNA
Salt Stress
ATTTTAATACGCGTGCGACGGTCGTAAG AGTATAGGTAATAGGTTATAATAAAATGATGGATATAATAT Unconverted DNA
ATTTTAATATGTGGACGTTGTACGAGTATAGGTAATAGCGTTAATAATAAAATGTATGATATAATATTAT Converted DNA
TL-2
Control
TTTTAAAATACGCGGTATCGTCGATCGTTTATGTTAGGGTGTAAAAAATTTATTTTGAAAATTTTTTTT Unconverted DNA
TTTAATAATGCGTGTTGTCGGCGTACGGGTAATAGCGTTAATAATAAAATGTATGGATATAATATTATT Converted DNA
Salt Stress
ATTTTAATACGCGTGCGACGGTCGTAAG AGTATAGGTAATAGGTTATAATAAAATGATGGATATAATAT Unconverted DNA
ATTTTAATATGTGGACGTTGTACGAGTATAGGTAATAGCGTTAATAATAAAATGTATGATATAATATTAT Converted DNA

(4)

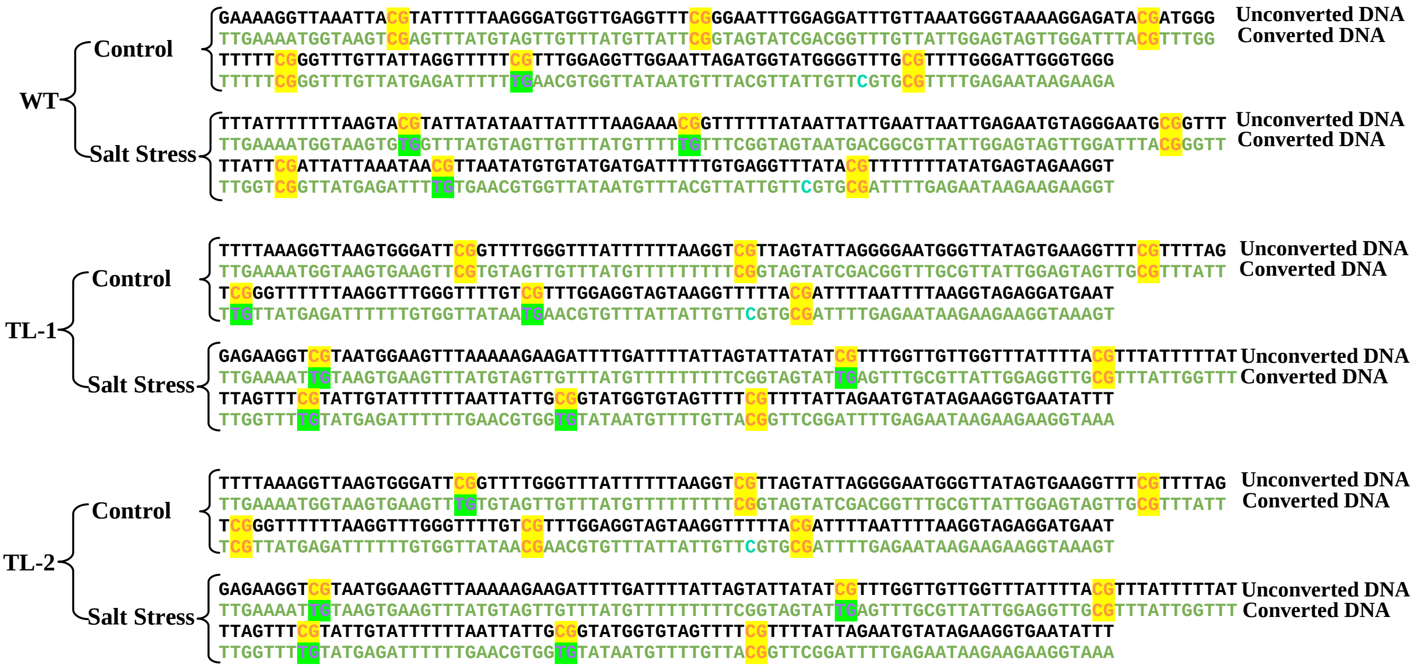
WT
Control
Salt Stress
TL-1
Control
Salt Stress
TL-2
Control
Salt Stress
Unconverted DNA
Converted DNA

(5)

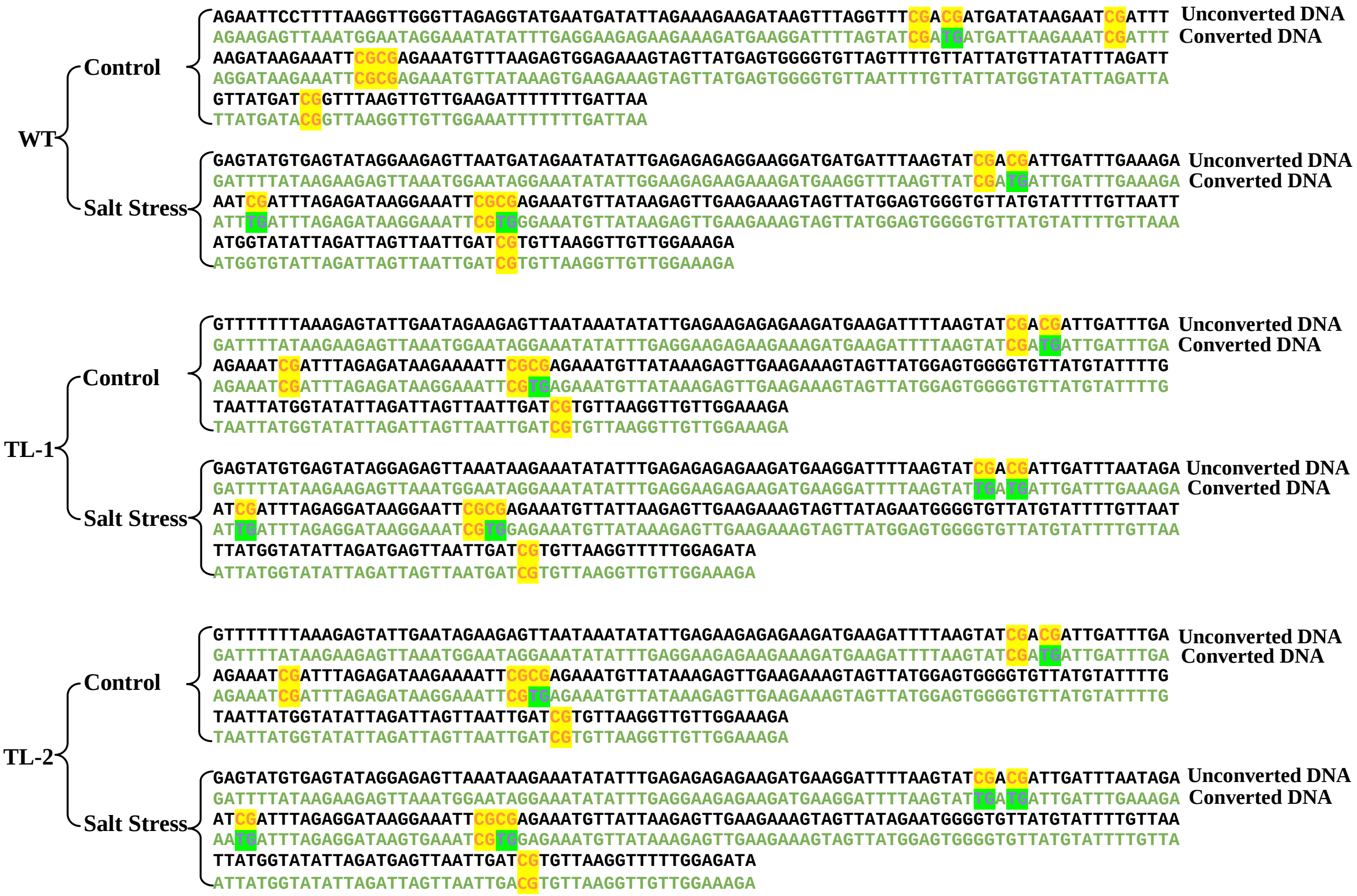
WT
Control
Salt Stress
TL-1
Control
Salt Stress
TL-2
Control
Salt Stress
Unconverted DNA
Converted DNA

(6)

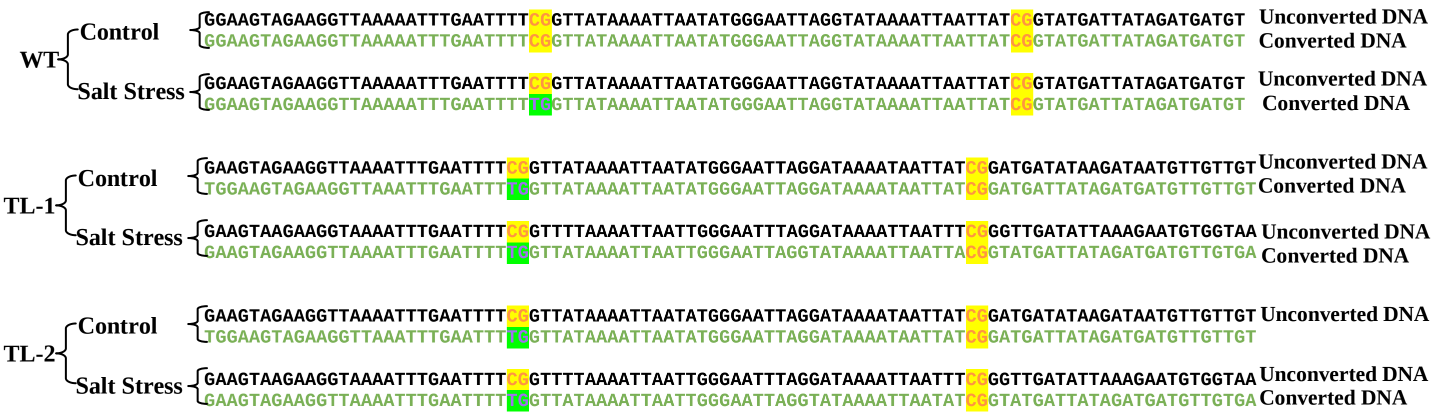

WT
Control
GGAAGTAGAAGGTTAAAAATTTGAATTTTCGGTTATAAAATTAATATGGGAATTAGGTATAAAATTAATTATCGGTATGATTATAGATGATGT Unconverted DNA
GGAAGTAGAAGGTTAAAAATTTGAATTTTCGGTTATAAAATTAATATGGGAATTAGGTATAAAATTAATTATCGGTATGATTATAGATGATGT Converted DNA
Salt Stress
GGAAGTAGAAGGTTAAAAATTTGAATTTTCGGTTATAAAATTAATATGGGAATTAGGTATAAAATTAATTATCGGTATGATTATAGATGATGT Unconverted DNA
GGAAGTAGAAGGTTAAAAATTTGAATTTTTGGTTATAAAATTAATATGGGAATTAGGTATAAAATTAATTATCGGTATGATTATAGATGATGT Converted DNA
TL-1
Control
GAAGTAGAAGGTTAAAATTTGAATTTTCGGTTATAAAATTAATATGGGAATTAGGATAAAATAATTATCGGATGATATAAGATAATGTTGTTGT Unconverted DNA
TGGAAGTAGAAGGTTAAATTTGAATTTTTGGTTATAAAATTAATATGGGAATTAGGATAAAATAATTATCGGATGATTATAGATGATGTTGTTGT Converted DNA
Salt Stress
GAAGTAAGAAGGTAAAATTTGAATTTTCGGTTTTAAAATTAATTGGGAATTTAGGATAAAATTAATTTCGGTTGATATTAAAGAATGTGGTAA Unconverted DNA
GAAGTAGAAGGTTAAAATTTGAATTTTTGGTTATAAAATTAATTGGGAATTAGGTATAAAATTAATTACGGTATGATTATAGATGATGTTGTGA Converted DNA
TL-2
Control
GAAGTAGAAGGTTAAAATTTGAATTTTCGGTTATAAAATTAATATGGGAATTAGGATAAAATAATTATCGGATGATATAAGATAATGTTGTTGT Unconverted DNA
TGGAAGTAGAAGGTTAAATTTGAATTTTTGGTTATAAAATTAATATGGGAATTAGGATAAAATAATTATCGGATGATTATAGATGATGTTGTTGT
Salt Stress
GAAGTAAGAAGGTAAAATTTGAATTTTCGGTTTTAAAATTAATTGGGAATTTAGGATAAAATTAATTTCGGTTGATATTAAAGAATGTGGTAA Unconverted DNA
GAAGTAGAAGGTTAAAATTTGAATTTTTGGTTATAAAATTAATTGGGAATTAGGTATAAAATTAATATCGGTATGATTATAGATGATGTTGTGA Converted DNA

(7)

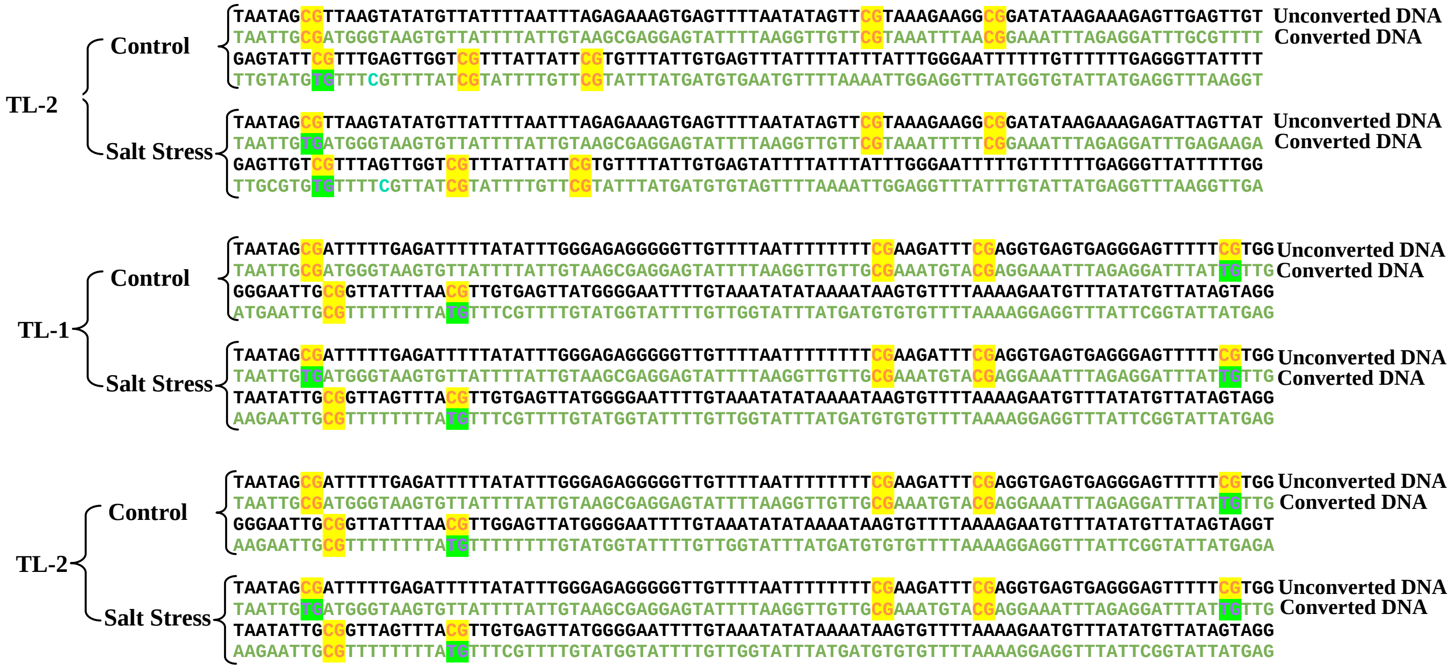

TL-2
Control
TAATAGCGTTAAGTATATGTTATTTTAATTTAGAGAAAGTGAGTTTTAATATAGTTCGTAAAGAAGGCGGATATAAGAAAGAGTTGAGTTGT Unconverted DNA
TAATTGCGATGGGTAAGTGTTATTTTATTGTAAGCGAGGAGTATTTTAAGGTTGTTCGTAAATTTAACGGAAATTTAGAGGATTTGCGTTTT Converted DNA
GAGTATTCGTTTGAGTTGGTCGTTTATTATTCGTGTTTATTGTGAGTTTATTTTATTTATTTGGGAATTTTTGTTTTTTGAGGGTTATTTT
TTGTATGTGTTTCGTTTTATCGTATTTTGTTCGTATTTATGATGTGAATGTTTTAAAATTGGAGGTTTATGGTGTATTATGAGGTTTAAGGT
Salt Stress
TAATAGCGTTAAGTATATGTTATTTTAATTTAGAGAAAGTGAGTTTTAATATAGTTCGTAAAGAAGGCGGATATAAGAAAGAGATTAGTTAT Unconverted DNA
TAATTGTGATGGGTAAGTGTTATTTTATTGTAAGCGAGGAGTATTTTAAGGTTGTTCGTAAATTTTTCGGAAATTTAGAGGATTTGAGAAGA Converted DNA
GAGTTGTCGTTTAGTTGGTCGTTTATTATTCGTGTTTTATTGTGAGTATTTTATTTATTTGGGAATTTTTGTTTTTTGAGGGTTATTTTTGG
TTGCGTGTGTTTTCGTTATCGTATTTTGTTCGTATTTATGATGTGTAGTTTTAAAATTGGAGGTTTATTTGTATTATGAGGTTTAAGGTTGA
TL-1
Control
TAATAGCGATTTTTGAGATTTTTATATTTGGGAGAGGGGGTTGTTTTAATTTTTTTTCGAAGATTTCGAGGTGAGTGAGGGAGTTTTTCGTGG Unconverted DNA
TAATTGCGATGGGTAAGTGTTATTTTATTGTAAGCGAGGAGTATTTTAAGGTTGTTGCGAAATGTACGAGGAAATTTAGAGGATTTATTGTTG Converted DNA
GGGAATTGCGGTTATTTAACGTTGTGAGTTATGGGGAATTTTGTAAATATATAAAATAAGTGTTTTAAAAGAATGTTTATATGTTATAGTAGG
ATGAATTGCGTTTTTTTTATTGTTTCGTTTTGTATGGTATTTTGTTGGTATTTATGATGTGTGTTTTAAAAGGAGGTTTATTCGGTATTATGAG
Salt Stress
TAATAGCGATTTTTGAGATTTTTATATTTGGGAGAGGGGGTTGTTTTAATTTTTTTTCGAAGATTTCGAGGTGAGTGAGGGAGTTTTTCGTGG Unconverted DNA
TAATTGTGATGGGTAAGTGTTATTTTATTGTAAGCGAGGAGTATTTTAAGGTTGTTGCGAAATGTACGAGGAAATTTAGAGGATTTATTGTTG Converted DNA
TAATATTGCGGTTAGTTTACGTTGTGAGTTATGGGGAATTTTGTAAATATATAAAATAAGTGTTTTAAAAGAATGTTTATATGTTATAGTAGG
AAGAATTGCGTTTTTTTTATTGTTTCGTTTTGTATGGTATTTTGTTGGTATTTATGATGTGTGTTTTAAAAGGAGGTTTATTCGGTATTATGAG
TL-2
Control
TAATAGCGATTTTTGAGATTTTTATATTTGGGAGAGGGGGTTGTTTTAATTTTTTTTCGAAGATTTCGAGGTGAGTGAGGGAGTTTTTCGTGG Unconverted DNA
TAATTGCGATGGGTAAGTGTTATTTTATTGTAAGCGAGGAGTATTTTAAGGTTGTTGCGAAATGTACGAGGAAATTTAGAGGATTTATTGTTG Converted DNA
GGGAATTGCGGTTATTTAACGTTGGAGTTATGGGGAATTTTGTAAATATATAAAATAAGTGTTTTAAAAGAATGTTTATATGTTATAGTAGGT
AAGAATTGCGTTTTTTTTATTGTTTTTTTTTGTATGGTATTTTGTTGGTATTTATGATGTGTGTTTTAAAAGGAGGTTTATTCGGTATTATGAGA
Salt Stress
TAATAGCGATTTTTGAGATTTTTATATTTGGGAGAGGGGGTTGTTTTAATTTTTTTTCGAAGATTTCGAGGTGAGTGAGGGAGTTTTTCGTGG Unconverted DNA
TAATTGTGATGGGTAAGTGTTATTTTATTGTAAGCGAGGAGTATTTTAAGGTTGTTGCGAAATGTACGAGGAAATTTAGAGGATTTATTGTTG Converted DNA
TAATATTGCGGTTAGTTTACGTTGTGAGTTATGGGGAATTTTGTAAATATATAAAATAAGTGTTTTAAAAGAATGTTTATATGTTATAGTAGG
AAGAATTGCGTTTTTTTTATTGTTTCGTTTTGTATGGTATTTTGTTGGTATTTATGATGTGTGTTTTAAAAGGAGGTTTATTCGGTATTATGAG

(8)

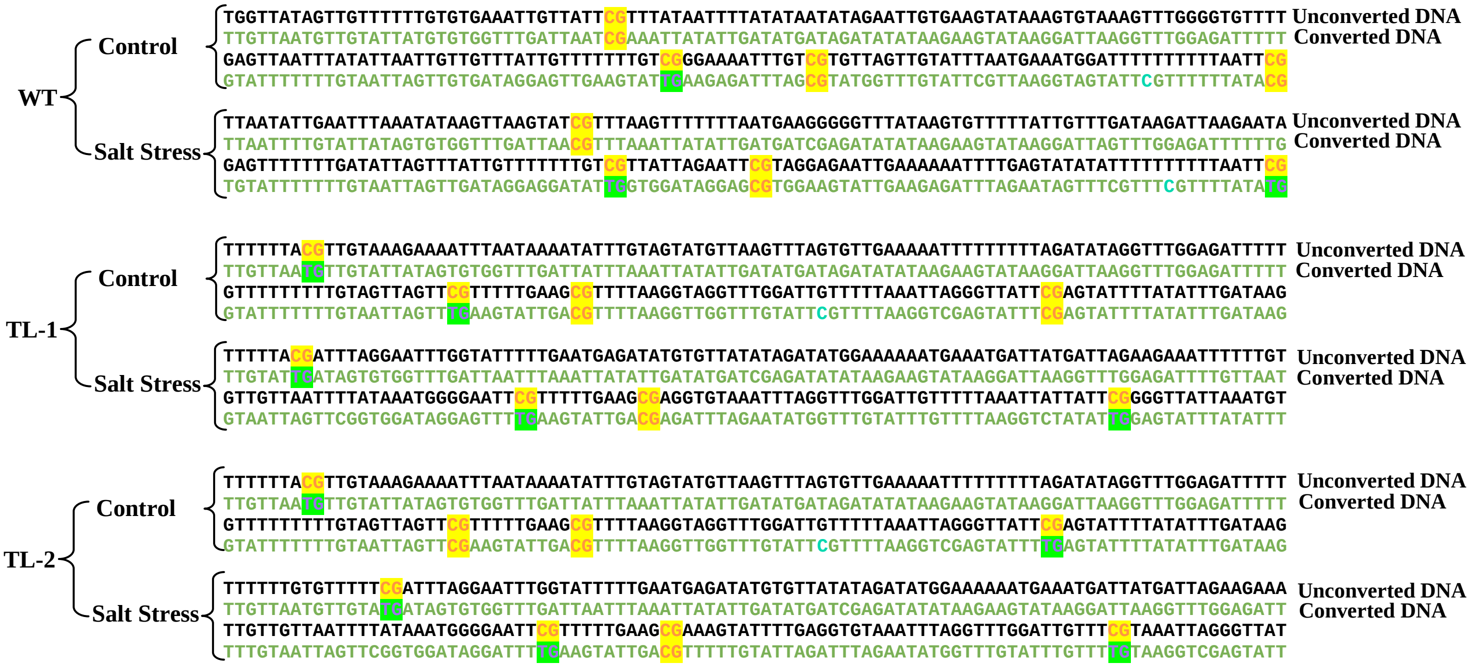
WT
Control
Salt Stress
TL-1
TL-2
Unconverted DNA
Converted DNA

(9)

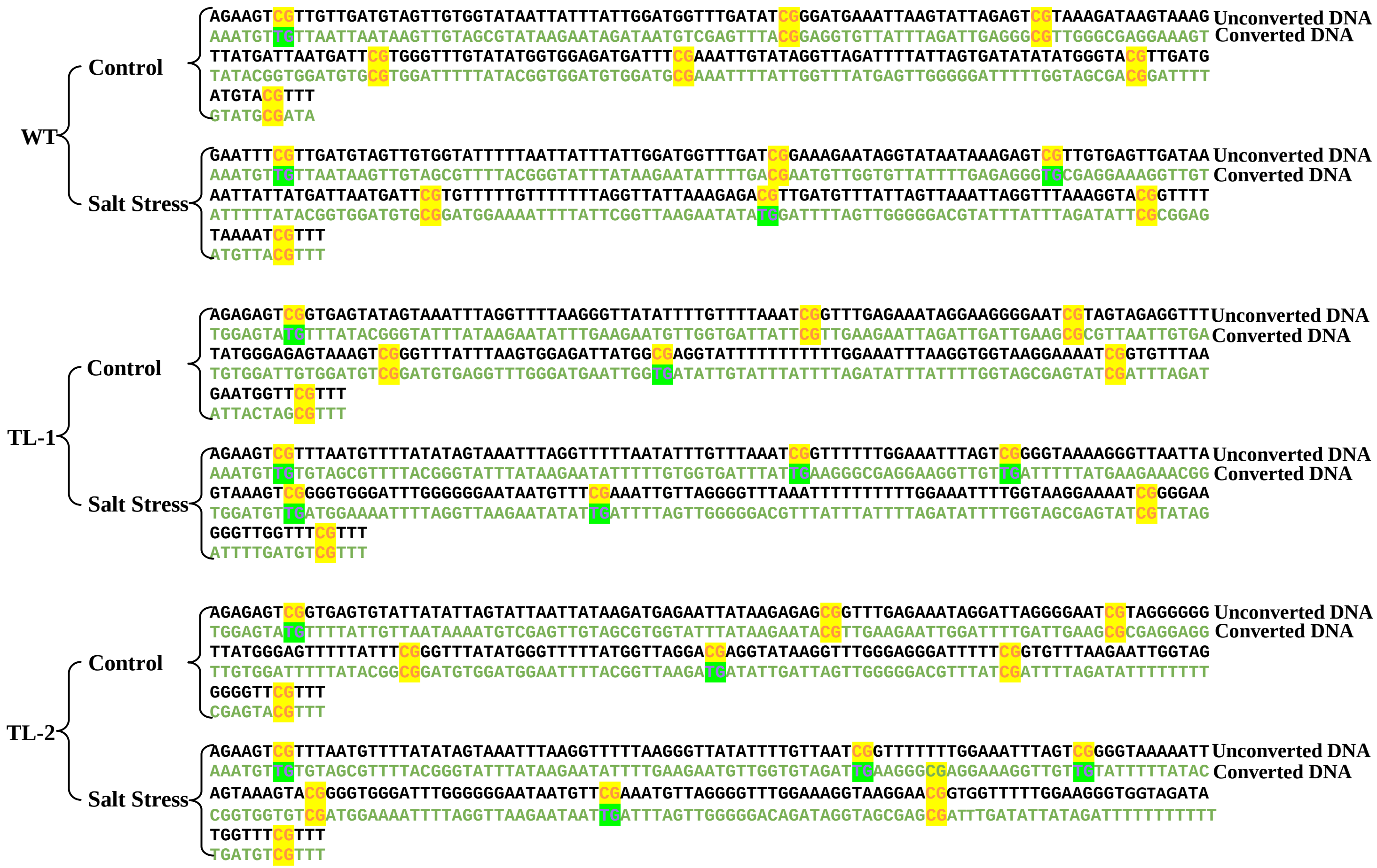
WT
Control
Salt Stress
TL-1
TL-2
Unconverted DNA
Converted DNA

(10)

Figure S2. Sequence alignment of bisulfite converted and unconverted DNA showing the methylation marks in the promoter regions of genes encoding enzymatic proteins of flavonoid biosynthetic and antioxidative pathways such as **(1)** Chalcone Synthase; **(2)** Chalcone Isomerase; **(3)** Flavanone 3-hydroxylase; **(4)** Flavonol Synthase; **(5)** Dihydroflavonol 4-reductase; **(6)** Anthocyanidin Synthase; **(7)** Glutathione S-transferase; **(8)** Ascorbate Peroxidase; **(9)** Glutathione Peroxidase and **(10)** Glutathione Reductase under control and salt stress (200mM NaCl) conditions in transgenic lines (TL-1) and (TL-2), respectively. **Control**: untreated wild type tobacco, TL-1 and TL-2 transgenic tobacco lines. **Salt Stress**: wild type and *AtROS1* transgenic lines (TL-1 and TL-2) treated with 200mM NaCl for 30 days. **Converted DNA**: Sequence of promoter region of target genes after bisulfite treatment. **Unconverted DNA**: Sequence of promoter region of target genes without bisulfite treatment. **WT**: wild type tobacco plants. **TL-1** and **TL-2**: *AtROS1* transgenic tobacco lines.

Table S1. Oligonucleotide sequences used for identification of *AtROS1* overexpressing transgenic tobacco

Primer Pair	Forward (5'-3')	Reverse (5'-3')	Product Size (bp)
R1	5'-AAGGTACTGAAGAAGTGGAGAG-3'	5'-TTTCTCTTGGGCGTGCTAGGA-3'	466
R2	5'- CGAGCAGCAGATGTTAAGGAAG-3'	5'-CAAACGCACTGGCAAAATGTCT-3'	520
Bar	5'-ATGAGACAAGCACGGTCAA-3'	5'-AAACCCACGTCATGCCAGTT-3'	300

Table S2. Oligonucleotide sequences used in relative transcript expression analysis of genes by quantitative Real-Time PCR

Gene	Forward (5'-3')	Reverse (5'-3')
CHS	5'-GGACTTGGCCGAAAACAACA-3'	5'-ACTATCCAAGTGGGTGTCATTGG-3'
CHI	5'-ATGCAGAGAGTCAGGCCATTG-3'	5'-GCCCCAACAGGTGATTGAGT-3'
F3H	5'-GGCTAAGCAACAAGATTTGGAGAA-3'	5'-TTTGGCAAGAACACACGCTAA-3'
FLS	5'-GGCCAATTTCTAAGCTGGTTAATG-3'	5'-GGTTTCTTCACTGAGGAAGCTTGT-3'
DFR	5'-GAGACTTGTCGACAGAAGCAGTTG-3'	5'-CTTGCATAGTTTTCAGCAGAAAGG-3'
ANS	5'-TGGCGTTGAAGCTCATACTGA-3'	5'-TGCCGTTACCCACTGTCCTT-3'
GST	5'-GCTTGCCTCATCGAGAAGGA-3'	5'-CAAAAGGATTGAGGGAAAGGTAAG-3'
APx	5'-CATGGCACTCTGCTGGTACCT-3'	5'-CCATTGTTTGCTCCATGTCCTT-3'
GPx	5'-GGTGGACAGGAGCCTGGAA-3'	5'-CACCATTCACATCAACCTTATCAAA-3'
GR	5'-TTTGCGATTGCAGTCAAAGC -3'	5'-GGGTACGCATGGTGACAAACT-3'
26S rRNA	5'-CACAATGATAGGAAGAGCCGAC-3'	5'-CAAGGGAACGGGCTTGGCAGAATC-3'

Table S3. Oligonucleotide sequences used for genome walking

Gene	Reverse primer for primary PCR (5'-3')	Reverse primer for secondary nested PCR (5'-3')
CHS	5'-TCTCAACTGTAAGCCCAGGCCCAAATC-3'	5'-ACACCCCATTCAAGCCCTTCACCAGTA-3'
CHI	5'-GGCACGTTGAGCCTCCCACATTACAT-3'	5'-TGCAACGTTGACAACATCAGGCTCAGT-3'
F3H	5'-GGGCTTGGTTTTCAACTTGGCCTTCTC-3'	5'-TGGGCTCGTCCATTACTGCCTTCTCTC-3'
FLS	5'-TCTTGAATTTGGGTGGATTGGCCTCAT-3'	5'-TTGGCCCTACTTCATGCTCTGATGGTG-3'
DFR	5'-AGGGGAATGGTTTCTTTGTCACGTCCA-3'	5'-CTGGCCATTTCTCTCGGACCATCTTTG-3'
ANS	5'-ATGATCTTCTCCTTTGGCGGCTCACA-3'	5'-CACACACTTTGCCGTTACCCACTGTCC-3'
GST	5'-GCCTGGCCAAAATATCAGCACACCAT-3'	5'-AGACAATTGCGCTTCGCTTTCCTTCAC-3'
APx	5'-CATGCCACCACTCCCAACTCTTCCTC-3'	5'-GGCGTCTTCATCCGCAGCATATTTCTC-3'
GPx	5'-CGATCGACGACGTTTCCTTCTTTGTCA-3'	5'-CCCCAAAGAACCCACCTTTGCTTGATT-3'
GR	5'-ATGAATGCACGGCAATCTCAGGTGAA-3'	5'-GGGGTACGCATGGTGACAAACTCCTCT-3'
Adaptor (F)	5'-GTAATACGACTCACTATAGGGC-3'	5'-ACTATAGGGCACGCGTGGT-3'

Table S4. Oligonucleotide sequences designed for bisulfite sequencing of promoters of genes encoding enzymes of flavonoid biosynthesis and antioxidative pathway

Gene	Forward (5'-3')	Reverse (5'-3')	Tm (°C)	No. of Cs present in amplified product	Product Size (bp)
CHS	5'-TTAAAGAATGGGGTTAGTTTAAGTTTAAA- 3'	5'-ACTATCCAAATAAATATCATTAAATCCAC-3'	57	14	284
CHI	5'-TGGTGAATTTTTTTGGTTTTATTAATATT-3'	5'-TCCTCTAAACTTTTACTTTTCCATTTAAC-3'	59	08	166
F3H	5'-ATTTTAAATAAGTTTTATAAGGGATGAAGA-3'	5'-AATTAAACATCAACACCATAATCAACTAC-3'	58	07	210
FLS	5'-TGGTGTGTTATTTTAAGTTTTAGTAATTGA-3'	5'-ACAATCACCTCTTTCTCCTCTTATAATAC-3'	59	05	195
DFR	5'-ATTGGTTTTTGGTTTGTTATGAGAT-3'	5'-AAATAACTTCATCAAAACTTCCTTCC-3'	59	06	178
ANS	5'-GAAGAAAGATGAAGGATTTTAAGTAT-3'	5'-ATAATTAACAAAATACATAACACCCCACT-3'	55	06	221
GST	5'-GGAAGTAGAAGGTTAAAAATTTGAATTTT-3'	5'-CTTCACAACAACATCATCTATAATCATAC-3'	58	02	103
APx	5'-TAATTGTTATGGGTAAGTGTTATTTTATTG-3'	5'-TCCTTACTCAACCTTAAACCTCATAATAC-3'	58	06	209
GPx	5'-TTGTTAATGTTGTATTATAGTGTGGTTTG -3'	5'-ACATCAACCTTATCAAATATAAAATACTC-3'	56	04	211
GR	5'-TGGAGTATTTTTATTGTTAATAAAAATGT-3'	5'-AAATCAAAAACAACATCAAAATCTATAAC -3'	56	07	248

Table S5. Oligonucleotide sequences for methylation specific PCR corresponding to methylated region of coding sequences of genes encoding enzymes of flavonoid biosynthesis and antioxidative pathway.

Gene	Forward (5’-3’)	Reverse (5’-3’)	T m (°C)	No. of Cs present in amplified product	Product Size (bp)
CHS	5’-TATTAAGTTATTCGGGTTTCGTTTATC- 3’	5’-TATCCAAATAAATATCATTAAATCCACG- 3’	59	08	191
CHI	5’-TGGAGTTTATTATTATTGAGAATTACGT- 3’	5’-CTAAACTTTTACTTTTCCATTTAACGAC- 3’	57	05	204
F3H	5’-GTTTTGTTTTTTGAGGAAAAATTAC- 3’	5’-AAAAAATAAATCACTATTTCACGCC- 3’	56	04	125
FLS	5’-GGTTGTAAGAGTATAGGTAATAGCGTTA- 3’	5’-AAACTTAAAATAACACACCATACAACGT- 3’	57	05	125
DFR	5’-TTTTATTTTATCGGTTGGAATTTTC- 3’	5’-TCTTTTTAACTACTTCCATTACGAC- 3’	55	04	170
ANS	5’-TTATTTTTTTTATTGCGTTTTTTTC- 3’	5’-ATAAATCCTCCTTACCTCCGAC- 3’	57	04	203
GST	5’-TGGGAATTAGGTATAAAATTAATTATCG- 3’	5’-ACCATACACTCACACGAAAACG- 3’	58	04	238
APx	5’-TATGGGTAAGTGTTATTTTATTGTAAGC- 3’	5’-CTCAACCTTAAACCTCATAATACCG- 3’	57	05	196
GPx	5’-AATTTAAATTATATTGATATGATCGA- 3’	5’-ATAAAATACTCGACCTTAAAACGAA- 3’	52	08	161
GR	5’-ATTGTTAATAAAAATGTCGAGTTGTAGC- 3’	5’-CCAAAAATATCTAAAATAAATAAACGTC- 3’	56	09	197

Table S6. Oligonucleotide sequences for methylation specific PCR corresponding to unmethylated region of coding sequences of genes encoding enzymes of flavonoid biosynthesis and antioxidative pathway.

Gene	Forward (5’-3’)	Reverse (5’-3’)	Tm (°C)	No. of Cs present in amplified product	Product Size (bp)
CHS	5’-TTAAGTTATTTGGGTTTTGTTTATTGG-3’	5’-ATCCAAATAAATATCATTAAATCCACAA-3’	59	08	188
CHI	5’-TGGAGTTTATTATTATTGAGAATTATGT-3’	5’-TCTAAACTTTTACTTTTCCATTTAACAAC-3’	55	05	205
F3H	5’-TTTGTTTTTTGAGGAAAAATTATGT-3’	5’-AAAAAATAAATCACTATTTCACACC-3’	55	04	123
FLS	5’-GGTTGTAAGAGTATAGGTAATAGTGTTA-3’	5’-AAAACTTAAAATAACACACCATACAACATT-3’	53	06	126
DFR	5’-TTATTTTATTGGTTGGAATTTTTGA-3’	5’-TTCTTTTTAACTACTTCCATTACAAC-3’	54	04	169
ANS	5’-ATTTTTTTTATTGTGTTTTTTTTGA-3’	5’-AACAATAAATCCTCCTTACCTCCA-3’	55	05	205
GST	5’-TGGGAATTAGGTATAAAATTAATTATTGG-3’	5’-CACCATACACTCACACAAAAACAC-3’	59	04	239
APx	5’-TGGGTAAGTGTTATTTTATTGTAAGTGA-3’	5’-TACTCAACCTTAAACCTCATAATACCAA-3’	58	06	196
GPx	5’-TGATTAATTTAAATTATATTGATATGATTG-3’	5’-AAAATACTCAACCTTAAAACAAA -3’	52	08	161
GR	5’-TATTGTTAATAAAAATGTTGAGTTGTAGTG-3’	5’-CCAAAAATATCTAAAATAAATAAACATC-3’	54	09	198
